# Nuclear import of SAMHD1 is mediated by a classical karyopherin α/β1 dependent pathway and confers sensitivity to Vpx_MAC_ induced ubiquitination and proteasomal degradation

**DOI:** 10.1186/1742-4690-11-29

**Published:** 2014-04-08

**Authors:** Torsten Schaller, Darja Pollpeter, Luis Apolonia, Caroline Goujon, Michael H Malim

**Affiliations:** 1 Department of Infectious Diseases, King’s College London, 2nd Floor, Borough Wing, Guy’s Hospital, London Bridge, London SE1 9RT, UK

**Keywords:** SAMHD1, Nuclear import, Karyopherin, Vpx, Macrophages, HIV-1, NLS, Ubiquitin linkage, Innate immunity, KPNA, KPNB, Impα, Impβ

## Abstract

**Background:**

The deoxynucleotide-triphosphate (dNTP) hydrolase sterile alpha motif domain and HD domain 1 (SAMHD1) is a nuclear protein that inhibits HIV-1 infection in myeloid cells as well as quiescent CD4 T-cells, by decreasing the intracellular dNTP concentration below a level that is required for efficient reverse transcription. The Vpx proteins of the SIV_SMM_/HIV-2 lineage of lentiviruses bind SAMHD1 and recruit an ubiquitin ligase, leading to polyubiquitination and proteasomal degradation.

**Results:**

Here, we have investigated the importance of nuclear localization for SAMHD1′s antiviral function as well as its sensitivity to the Vpx protein of SIV_MAC_. Using GST pull down assays, as well as RNA silencing approaches, we show that SAMHD1 preferentially uses karyopherin α2 (KPNA2) and a classical N-terminal nuclear localization signal (^14^KRPR^17^) to enter the nucleus. Reduction of karyopherin β1 (KPNB1) or KPNA2 by RNAi also led to cytoplasmic re-distribution of SAMHD1. Using primary human monocyte-derived macrophages (MDM), a cell type in which SAMHD1 is naturally expressed to high levels, we demonstrate that nuclear localization is not required for its antiviral activity. Cytoplasmic SAMHD1 still binds to Vpx_MAC_, is efficiently polyubiquitinated, but is not degraded. We also find that Vpx_MAC_-induced SAMHD1 degradation was partially reversed by ubiquitin carrying the K48R or K11R substitution mutations, suggesting involvement of K48 and K11 linkages in SAMHD1 polyubiquitination. Using ubiquitin K-R mutants also revealed differences in the ubiquitin linkages between wild type and cytoplasmic forms of SAMHD1, suggesting a potential association with the resistance of cytoplasmic SAMHD1 to Vpx_MAC_ induced degradation.

**Conclusions:**

Our work extends published observations on SAMHD1 nuclear localization to a natural cell type for HIV-1 infection, identifies KPNA2/KPNB1 as cellular proteins important for SAMHD1 nuclear import, and indicates that components of the nuclear proteasomal degradation machinery are required for SAMHD1 degradation.

## Background

Host cells have evolved a number of antiviral factors that must be overcome by retroviruses to establish a productive infection. Intriguingly, early events in human immunodeficiency virus 1 (HIV-1) infection including uncoating, the liberation of the viral genome, as well as reverse transcription are vulnerable targets for restriction factors such as members of the APOBEC3 family, TRIM5α and TRIMCyp, and the recently identified sterile alpha motif (SAM) and HD domain containing 1 (SAMHD1), a dNTPase that inhibits HIV-1 in differentiated myeloid cells, as well as resting CD4+ T-cells [1-9]. SAMHD1 is targeted for proteasomal degradation by the lentiviral accessory proteins Vpx or Vpr, which recruit a cellular ubiquitin ligase complex. The activity of Vpx/Vpr from diverse primate immunodeficiency viruses is species-specific, and HIV-1 Vpr proteins do not counteract human SAMHD1 [10,11]. As a result, HIV-1 infects cells of myeloid origin, such as dendritic cells or macrophages, with limited efficiency and addition of Vpx_MAC_ containing virus like-particles relieves this block [12]. Intriguingly, HIV-1 infection of dendritic cells in the presence of Vpx causes dendritic cell activation and immune activation through an unknown sensor resulting in cytokine production, suggesting that infection of dendritic cells *in vivo* may be disadvantageous for sustained virus infection [13].

Mutations in SAMHD1 have been associated with Aicardi-Goutières syndrome (AGS) a condition presenting with increased type I interferon levels mimicking congenital viral infection [14,15]. Wild type SAMHD1 is localized to the nucleus, while AGS causing mutations can disrupt nuclear localization leading to SAMHD1 accumulation in the cytoplasm [15,16]. Recently, three independent groups have identified the nuclear localization signal (NLS) of human SAMHD1, and have demonstrated that disruption of this N-terminal motif results in cytoplasmic accumulation [17-19]. Hofmann et al. proposed that Vpx_MAC_ triggers SAMHD1 degradation specifically in the nucleus [18], while, in contrast, Laguette et al. proposed that nuclear export of SAMHD1 is required for its degradation by Vpx_MAC_[10]. In addition, Brandariz-Nuniz et al. suggested that Vpx_HIV-2/2B_ can degrade cytoplasmic SAMHD1 [17], which could not be confirmed by Hofmann et al. [18]. The identification of determinants leading to resistance of cytoplasmic SAMHD1 to Vpx_MAC_ mediated degradation may therefore help to address these discrepancies.

The nuclear import of cargo is mediated through diverse pathways involving the action of karyopherins, a group of at least 20 proteins in humans [20]. While karyopherin β (KPNB, importin β) family members can directly interact with some NLSs, they commonly engage their cargo indirectly through the recruitment of proteins of the karyopherin α (KPNA/importin α) family, of which there are at least 7 different members in human [21]. Karyopherin α proteins can bind a diversity of NLSs including monopartite NLSs, consisting of a single cluster of basic amino acids, bipartite NLSs consisting of multiple clusters as well as additional nonclassical NLSs [22]. The selection of nuclear import pathways for a particular cargo may vary, and specific NLS-KPNA interactions have been shown to be dependent on the cell type, as well as stages of cellular development or differentiation [23-27]. A recent report by Guo et al. employed co-immunoprecipitation experiments to investigate interaction between KPNB1 and SAMHD1, however systematic functional analyses of the importance of karyopherin α proteins or KPNB1 in SAMHD1 nuclear import have not been performed [28].

Here we have extended the characterization of SAMHD1 nuclear import requirements to primary monocyte-derived macrophages (MDM), a natural target cell for HIV-1, and have addressed the mechanism of resistance of cytoplasmic SAMHD1 to Vpx_MAC_ induced degradation. We have confirmed the NLS in human SAMHD1 and show that SAMHD1 is imported into the nucleus through a classical nuclear import pathway involving the cellular proteins karyopherin α2 (KPNA2) as well as karyopherin β1 (KPNB1). Depletion of either protein through RNAi results in a partial cytoplasmic redistribution of SAMHD1 and mutational inactivation of the NLS disrupts SAMHD1 binding to KPNA2. Consistent with observations made using cell lines, our data in MDM demonstrate that cytoplasmic SAMHD1 is still able to inhibit HIV-1 infection and is less sensitive to Vpx_MAC_ induced degradation. Interestingly, in MDM a substantial amount (~20%) of SAMHD1 NLS mutant localizes to the nucleus, suggesting either co-operative import with endogenous wild type SAMHD1 or the use of alternative import ways independent of the N-terminal NLS. We show that Vpx_MAC_ and Vpx_HIV-2/ROD_ both interact with SAMHD1 mutated in its NLS, and that wild type as well as NLS mutant SAMHD1 are targets for Vpx_MAC_ dependent mono- and polyubiquitination. These observations imply that the reduced sensitivity of cytoplasmic SAMHD1 to Vpx_MAC_ mediated degradation, as compared to nuclear SAMHD1, is not attributable to reduced binding of SAMHD1 to Vpx_MAC_ or by lower levels of SAMHD1 ubiquitination. Using specific ubiquitin lysine to arginine mutants we demonstrate differences in the ubiquitin linkage patterns between nuclear and cytoplasmic SAMHD1, suggesting a connection to the resistance of cytoplasmic SAMHD1 to Vpx_MAC_ induced degradation. These findings suggest that specific components of the nuclear proteasomal machinery are required for Vpx_MAC_ induced SAMHD1 degradation.

## Results

### The N-terminus of SAMHD1 comprises a classical NLS

**Figure 1:**
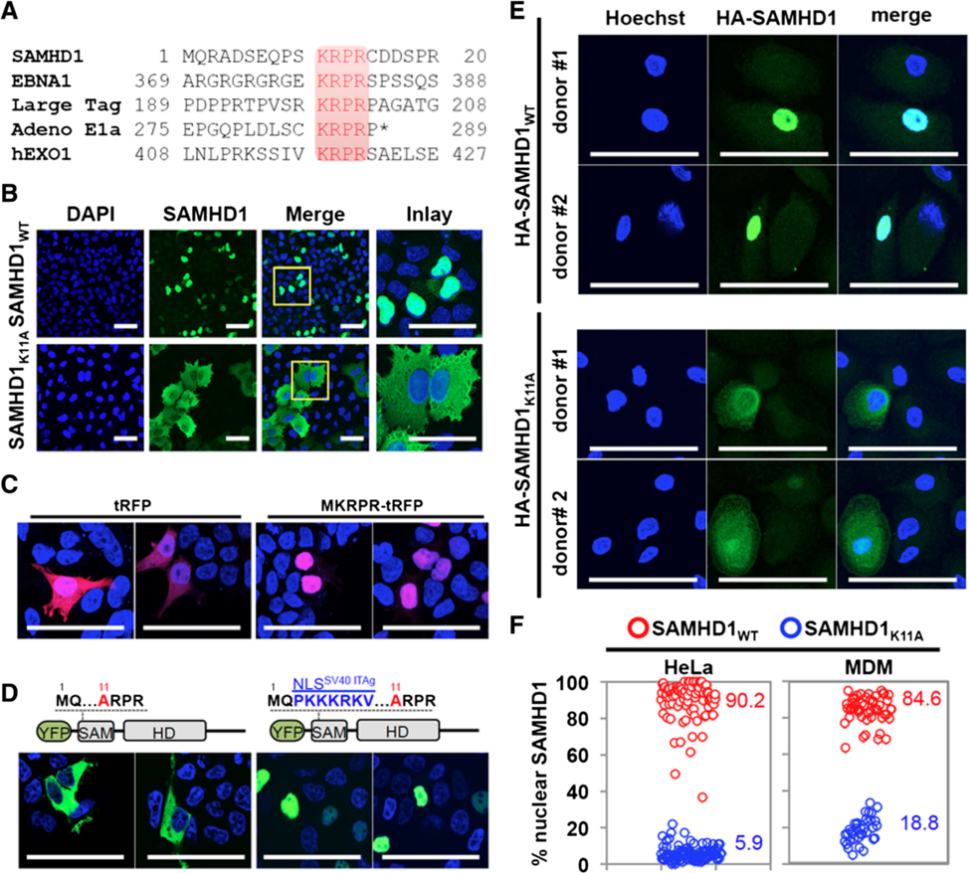
**Determination of the SAMHD1 nuclear localization signal (NLS). A)** Comparison of the SAMHD1 amino acid sequence with known nuclear localization signals (NLS) from EBNA1, polyoma virus large T antigen, adenovirus E1a and human EXO1 identifies an N-terminal four amino acid stretch as a putative NLS. **B)** Substitution of a critical lysine residue K11 with alanine abrogates SAMHD1 nuclear localization and causes cytoplasmic accumulation. HeLa cells transduced with HA-SAMHD1_WT_ or HA-SAMHD1_K11A_ expressing MLV vectors were subjected to immunofluorescence using a HA-specific antibody and nuclei were stained with DAPI. **C)** Expression of turbo red fluorescence protein (tRFP) fused to amino-terminal KRPR sequence in 293 T cells causes redistribution of the protein from a pan-cellular to a nuclear localization. Nuclei were stained with DAPI and tRFP was detected by autofluorescence. **D)** Fusion of the SV40 large T antigen NLS is sufficient to rescue nuclear localization of YFP-SAMHD1_K11A_. 293 T cells were transfected with YFP-SAMHD1_K11A_ (left panels) or YFP-SAMHD1_K11A_ fused to the NLS of the SV40 large T antigen (right panels) and autofluorescence was measured 48 h after transfection. Nuclei were stained with DAPI. **E)** Primary monocyte-derived macrophages from two independent donors were transduced with HA-tagged wild type or K11A mutant SAMHD1 expressing HIV-1 lentiviral vectors and HA-immunostaining was performed to detect SAMHD1 three days later. **F)** Quantification of the percentage of nuclear SAMHD1 for HA-SAMHD1_WT_ and HA-SAMHD1_K11A_ expressing cells comparing HeLa cells with primary monocyte derived macrophages (MDM). Each circle represents one cell and numbers indicate average percentages of cells shown. Scale bars, 50 μm.

SAMHD1 is a nuclear protein [15], however the pathway used for nuclear import, and in particular the identity of the cellular import receptors, is largely unexplored. We interrogated the SAMHD1 amino acid sequence to identify potential nuclear localization signals (NLSs) and found a stretch of four amino acids ^11^KRPR^14^ in the SAMHD1 amino terminus that, firstly, is predicted by the motif-finding online program PSORTII (http://psort.nibb.ac.jp) as a putative NLS and, secondly, has been shown to mediate the nuclear import of proteins such as Epstein-Barr virus nuclear antigen 1 (EBNA1), polyoma large T antigen, adenovirus E1a as well as human exonuclease 1 (EXO1) (Figure 1A). We therefore examined the consequences of changing the lysine residue at position 11 to alanine (SAMHD1_K11A_). This substitution resulted in impaired nuclear import and the re-distribution of SAMHD1 to the cytoplasm, as judged by immunofluorescence experiments in HeLa cells expressing wild type or K11A mutant forms of SAMHD1 (Figure 1B and F). Whereas 90.2% of wild type SAMHD1 cellular protein was localized to the nucleus, 94.1% of SAMHD1_K11A_ was localized to the cytoplasm homogenously across the cell population (Figure 1F). A similar result was obtained when THP-1 cells that naturally express SAMHD1 were transduced with MLV vectors encoding YFP-tagged wild type SAMHD1 or the K11A mutant (Additional file 1: Figure S1). To confirm that KRPR can function as an autonomous NLS, we expressed red fluorescence protein (RFP) fusion with KRPR at the amino-terminus. In contrast to RFP, which was present in both the nucleus and the cytoplasm, KRPR-RFP was exclusively found in the nucleus (Figure 1C). Next, we expressed a protein consisting of the SAMHD1_K11A_ mutant fused to a heterologous NLS from SV40 large T antigen (amino acid sequence PKKKRKV). SAMHD1_K11A_-SV40NLS was found exclusively in the nucleus, suggesting that import was restored by the SV40 large T antigen NLS (Figure 1D). To demonstrate that ^11^KRPR^14^ also serves as an NLS in primary cells, MDM were transduced with HA-SAMHD1 or HA-SAMHD1_K11A_ expressing lentiviral vectors. Ectopic expression of HA-SAMHD1_WT_ in MDM resulted in nuclear accumulation of SAMHD1 (Figure 1E). As expected, the K11A mutant predominantly localized to the cytoplasm (Figure 1E), however ~20% of HA-SAMHD1_K11A_ was also found in the nucleus, suggestive of cooperative nuclear import with endogenous SAMHD1 as has been proposed for other proteins [29] or for nuclear import independent of the N-terminal NLS (Figure 1E and F).

These results indicate that the N-terminal peptide ^11^KRPR^14^ functions as the major NLS of human SAMHD1 in both cell lines as well as MDM, consistent with recently published results [17-19].

### Karyopherin α members and karyopherin β1 (KPNB1) mediate SAMHD1 nuclear import

**Figure 2:**
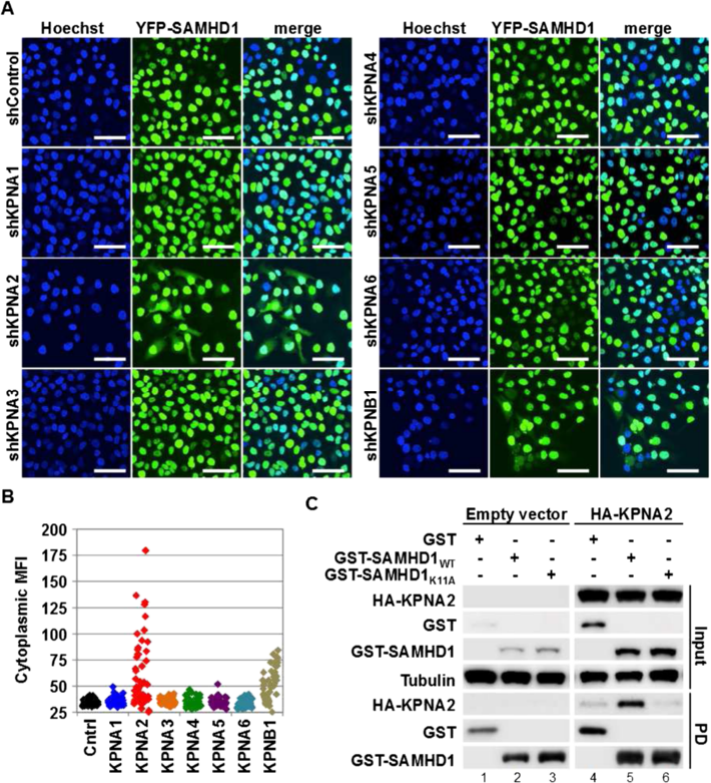
**SAMHD1 nuclear import is mediated by KPNA2 and KPNB1. A)** HeLa cells stably transduced with an MLV-vector encoding YFP-tagged wild type SAMHD1 were transiently transduced with Retro-SIREN-Q (Clontech) shRNA encoding vectors expressing two independent shRNAs for each karyopherin or shControl and subjected to autofluorescence microscopy 48 h after transduction. Nuclei were stained with Hoechst. All pictures were generated using the same microscope settings and analyzed using the same conditions. Scale bars, 50 μm. **B)** Cytoplasmic fluorescence was determined using software ImageJ for thirty cells for each slide shown in A) in an area of 535 pixels (total image size 1024x1024 pixels) of cytoplasm in proximity of the nucleus for each cell. Each dot represents measurement of cytoplasmic mean fluorescence intensities (MFIs) for one cell. Each cell was only counted once. **C)** GST-tagged wild type or SAMHD1_K11A_ encoding plasmids or the GST control were co-transfected with empty vector or HA-KPNA2 encoding vector in 293 T cells and GST-pull down (PD) was performed 48 h later. Tubulin served as the input loading control.

Karyopherin β1 (KPNB1) has recently been identified in SAMHD1 co-immunoprecipitation experiments [28]. To investigate the functional role of KPNB1 and to address whether cellular karyopherin α proteins are involved in the nuclear import of SAMHD1 and can bind to the N-terminal NLS of SAMHD1, we first transduced HeLa cells expressing YFP-tagged SAMHD1 with MLV-vectors encoding shRNAs targeting the individual cellular karyopherin αs 1 to 6 as well as karyopherin β1. Each karyopherin was targeted with two independent shRNAs, which efficiently and specifically reduced the different karyopherins as confirmed by matching co-transfection assays with HA-tagged KPNAs and western blot analysis (Additional file 1: Table S1 and Figure S2A). Transduction with vectors encoding shRNAs targeting KPNA2 but not karyopherin α members KPNA1, KPNA3, KPNA4, KPNA5 or KPNA6 was sufficient to cause partial cytoplasmic redistribution of SAMHD1 as judged by the determination of the mean fluorescence intensity in the cytosolic area (using ImageJ software) (Figure 2A and B).

To verify KPNA2 binding to the identified N-terminal NLS in SAMHD1, we performed GST-pull down assays using GST-SAMHD1 or GST-SAMHD1_K11A_ and HA-tagged KPNA2. We found that wild type SAMHD1 but not SAMHD1_K11A_ interacted with HA-KPNA2 (Figure 2C, lane 5 vs. 6), suggesting that KPNA2 is recruited to the N-terminal NLS. We note that in cells expressing KPNA2 specific shRNAs, SAMHD1 was only partially redistributed to the cytoplasm, possibly pointing to a level of redundancy in usage of karyopherin α members. Performing GST pull down assays with HA-KPNA1, 3, 4, 5, or 6 suggested that KPNA1 and KPNA6 might also interact with SAMHD1. In both cases, however, substantial binding to GST-SAMHD1_K11A_ was also seen (Additional file 1: Figure S3, lanes 23 and 28) implying either that detected binding represents background, or that karyopherin α interactions with other motifs within SAMHD1 may occur. Although it is possible that such interactions may underlie the low levels of SAMHD1_K11A_ nuclear accumulation seen in some conditions (Figure 1E and F), their contributions to the overall nuclear import of SAMHD1 are likely to be of minor importance.

Karyopherin β1 (KPNB1) has been demonstrated to target KPNA2 bound cargo for nuclear import [30,31]. We therefore investigated whether KPNB1 reduction would also lead to cytoplasmic re-distribution of SAMHD1 in HeLa cells. Knock-down of KPNB1 led to a substantial increase in the cytoplasmic mean fluorescence intensity (Figure 2B). Similar results were obtained in 293 T cells by western blot analysis detecting endogenous KPNB1, demonstrating moderate efficiency of KPNB1 reduction (Additional file 1: Figure S2B). Together these data suggest that KPNA2 is the predominant but possibly not the only karyopherin α that binds to the SAMHD1 NLS and that KPNB1 is also important for SAMHD1 nuclear import, indicating utilization of a classical KPNA/KPNB1 nuclear import pathway.

### Cytoplasmic SAMHD1 is less sensitive to Vpx_MAC_ induced degradation

**Figure 3:**
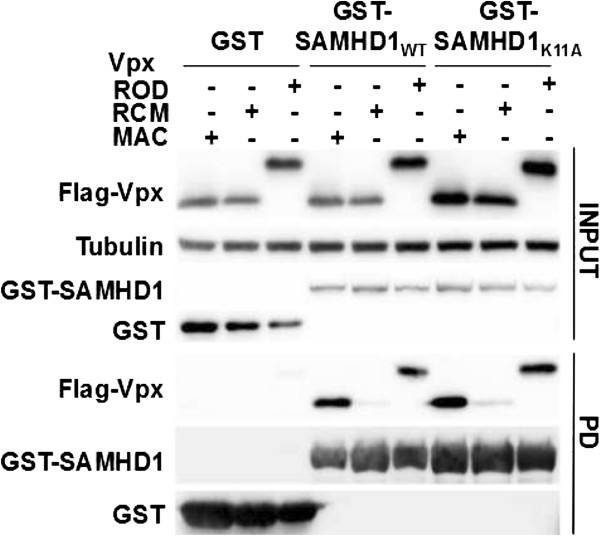
**SAMHD1**_**WT **_**and SAMHD1**_**K11A **_**bind Vpx**_**MAC **_**with similar efficiencies.** 293 T cells were co-transfected with plasmids expressing GST, GST-SAMHD1 or GST-SAMHD1_K11A_ along with plasmids expressing Flag-tagged Vpx derived from HIV-2_ROD_, SIV_MAC_ or SIV_RCM_. GST-pull down was performed and samples were separated using 15% SDS-PAGE gels. Vpx proteins were detected using an anti-Flag antibody, and SAMHD1 was detected using an anti-GST antibody. Tubulin served as a loading control for the input.

Human SAMHD1 activity is counteracted by the Vpx protein from SIV_MAC251_ but not from SIV_RCM_[6]. Vpx engages the CUL4A-DDB1 RING ubiquitin ligase complex through interaction with DCAF1, leading to ubiquitination and proteasomal degradation of its binding partners [32-34]. We tested whether cytoplasmic SAMHD1_K11A_ interacts with Vpx_MAC_ using a GST-pull down assay. GST-tagged wild type or K11A mutant SAMHD1 both interacted with Flag-tagged Vpx_MAC_ or Vpx_HIV-2/ROD_, but not with Vpx_RCM_ (Figure 3). This confirms that Vpx_MAC_ and Vpx_HIV-2/ROD_ can interact with SAMHD1 in the nucleus or cytoplasm, as demonstrated previously [18].

**Figure 4:**
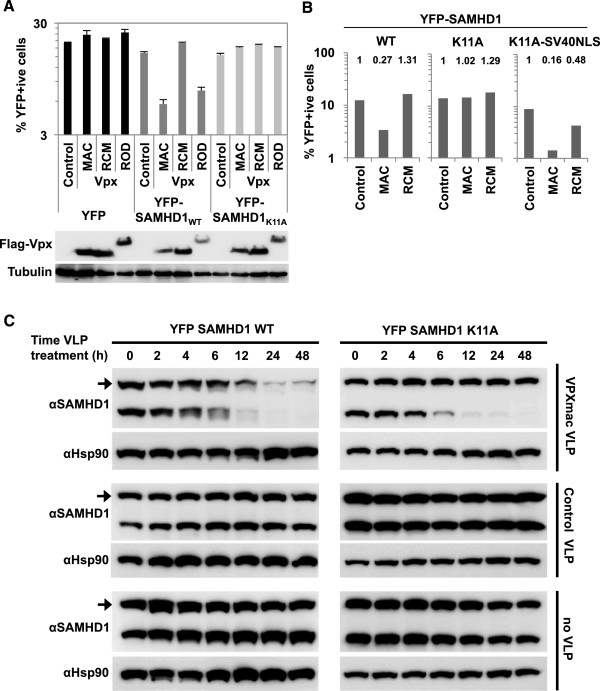
**SAMHD1**_**K11A **_**has reduced sensitivity to Vpx**_**MAC **_**mediated degradation. A)** 293 T cells were transfected with plasmids encoding YFP, YFP-SAMHD1_WT_, or YFP-SAMHD1_K11A_ along with plasmids encoding for Flag-tagged Vpx proteins from HIV-2_ROD_, SIV_MAC_ or SIV_RCM_ as indicated and percentages of YFP-positive cells were determined 24 h after transfection. A parallel sample was used for western blot analysis using an anti-Flag antibody (lower panel). Tubulin served as a loading control. **B)** YFP-tagged SAMHD1_WT_, SAMHD1_K11A_ or SAMHD1_K11A_-SV40NLS were co-transfected in parallel with Flag-Vpx expression plasmids as in A) and percentages of YFP + cells were measured 24 h post transfection. Fold changes are indicated above the bars. **C)** Time course of Vpx_MAC_ VLP or control VLP treatment of THP-1 cells expressing YFP-SAMHD1_WT_ or YFP-SAMHD1_K11A_. Western blot analysis was performed with an anti-SAMHD1 antibody detecting endogenous SAMHD1 as well as ectopically expressed YFP-SAMHD1 (arrow). Hsp90 served as loading control.

We next tested whether SAMHD1_K11A_ is sensitive to Vpx_MAC_ induced degradation. 293 T cells were co-transfected with YFP-tagged wild type or K11A mutant SAMHD1 encoding plasmids or with an YFP control plasmid, together with plasmids expressing Vpx_RCM_, Vpx_MAC_ or Vpx_HIV-2/ROD_. At 24 h, the percentages of YFP-positive cells were enumerated by flow cytometry. Expression of Vpx_MAC_ or Vpx_HIV-2/ROD_, but not Vpx_RCM_, reduced the percentage as well as the mean fluorescence intensity (MFI) of wild type YFP-SAMHD1 positive cells, whereas YFP-SAMHD1_K11A_ was unaffected by expression of any Vpx protein (Figure 4A and Additional file 1: Figure S4). We confirmed expression of the transfected Vpx proteins by western blot using an anti-Flag antibody. Importantly, YFP-SAMHD1_K11A_-SV40NLS regained sensitivity to Vpx_MAC_ induced degradation (Figure 4B). Next, we aimed to recapitulate these observations in cells responding to Vpx_MAC_ VLP treatment and generated THP-1 cells expressing YFP-tagged SAMHD1_WT_ or SAMHD1_K11A_ to similar levels as the endogenous SAMHD1 (Figure 4C). Treatment of YFP-SAMHD1_WT_ but not YFP-SAMHD1_K11A_ expressing THP-1 cells caused a substantial reduction of the YFP-tagged SAMHD1 by 12 h after VLP treatment. Of note, the endogenous SAMHD1 was depleted with similar kinetics in both cell lines, and more rapidly than the tagged YFP-SAMHD1_WT_ protein. We next compared the stabilities of YFP-tagged and HA-tagged SAMHD1 in the absence of Vpx in S^35^ pulse chase experiments in 293 T cells and observed an increased stability for YFP-tagged SAMHD1, which likely explains the reduced kinetics in degradation in THP-1 cells as compared to endogenous SAMHD1 (Additional file 1: Figure S5). However, and most importantly, Vpx_MAC_ VLP treatment specifically reduced endogenous as well as ectopically expressed wild type but not NLS mutant SAMHD1 validating co-transfection results from 293 T (Figure 4). To exclude the possibility that SAMHD1_K11A_ has greater inherent stability we analysed HA- or YFP-tagged wild type or K11A mutant SAMHD1 by S^35^ pulse chase experiments and found that the mutation had modest or no effect on stability in the absence of Vpx_MAC_ (Additional file 1: Figure S5). We conclude that although cytoplasmic SAMHD1 interacts with Vpx_MAC_ and Vpx_HIV-2/ROD_, degradation in response to these Vpx proteins requires SAMHD1 nuclear localization, as shown in other studies [10,17,18].

To exclude the possibility that residue K11 in SAMHD1 is a target site for ubiquitination and that substitution of this residue with alanine caused resistance to Vpx_MAC_ mediated degradation by disrupting a possible ubiquitnation target site, we analysed naturally occurring variations of the NLS in SAMHD1 sequences from other species for their impact on subcellular localization as well as sensitivity to Vpx_MAC_ induced degradation (Additional file 1: Figure S6A). We therefore replaced the KRPR motif in YFP-tagged human SAMHD1 with KRSR (*Rattus norvegicus*), KRLR (*Chlorocebus pygerythrus, Erythrocebus patas*), KRAR (*Echinops telfairi, Monodelphis domestica, Gallus gallus, Macropus eugenii*), KKCR (*Pteropus vampyrus*), NRPR (*Erinaceus europaeus*), KRLH (*Chlorocebus tantalus*), KRPG (*Oryctolagus cuniculus*) or KRAC (*Anolis carolinensis*). We first investigated the subcellular localization of the individual YFP-tagged SAMHD1 variants and found that four of the eight variations (NRPR, KRLH, KRPG and KRAC) were distributed mainly in the cytoplasm, whereas the other variants (KRSR, KRLR, KRAR and KKCR) were found predominantly in the nucleus (Additional file 1: Figure S6B).

Next, we investigated the sensitivity of these SAMHD1 chimerae to Vpx_MAC_ induced degradation. SAMHD1 NLS variants that localized predominantly to the cytoplasm (NRPR, KRLH, KRPG and KRAC) were mainly insensitive to Vpx_MAC_ mediated degradation, whereas variants that were found predominantly in the nucleus (KRSR, KRLR, KRAR and KKCR) were sensitive, further supporting the conclusion that nuclear localization of SAMHD1 is a prerequisite for Vpx_MAC_ initiated degradation (Additional file 1: Figure S6C). Our results suggest that K11 and R14 are critical for the nuclear import of human SAMHD1, and that sensitivity to Vpx_MAC_ induced degradation depends on SAMHD1 nuclear localization, as suggested by parallel studies [17,18].

### Cytoplasmic SAMHD1 retains antiviral activity against HIV-1

**Figure 5:**
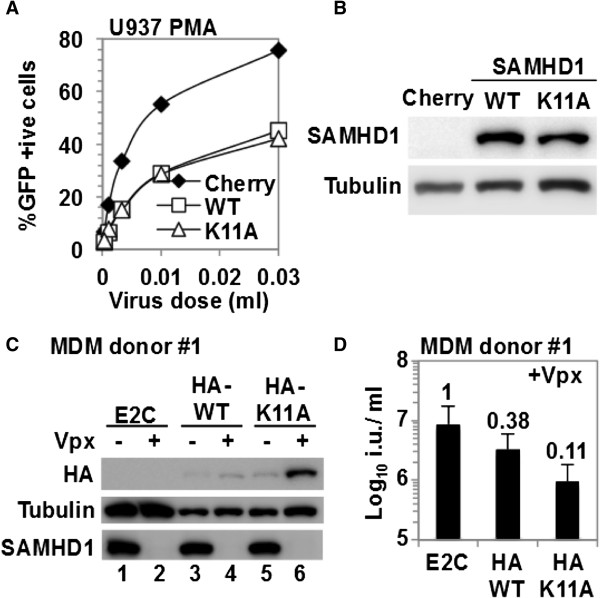
**Wild type SAMHD1 as well as SAMHD1**_**K11A **_**inhibit HIV-1 infection in cell lines and primary monocyte derived macrophages. A)** U937 cells transduced with lentiviral vectors expressing wild type or K11A mutant SAMHD1, or mCherry respectively, were differentiated with 100 ng/ml PMA for 2 days and then infected with an HIV-1 YFP reporter vector at different doses. Infected cells were enumerated by flow cytometry 48 h after infection. A representative of at least three independent experiments is shown. **B)** A parallel sample to A) was used for western blotting using a SAMHD1 specific antibody. Tubulin served as a loading control. **C)** Monocyte-derived macrophages were transduced with lentiviral vectors encoding HA-tagged SAMHD1_WT_ or SAMHD1_K11A_ in the presence or absence of virus like particles delivering Vpx_MAC_ and cells were used for western blotting using anti-HA, anti-SAMHD1 as well as anti-tubulin antibodies. Cells transduced with a lentivirus vector expressing E2-Crimson (E2C) served as a control. **D)** A parallel sample of C) was used to test the infectivity of an HIV-1 GFP reporter virus which was measured 48 h post infection by flow cytometry. Infectious titers (i.u./ml) were calculated from three independent viral doses. Numbers above bars present fold changes compared to the E2C control.

To investigate whether SAMHD1′s antiviral activity is dependent on its nuclear localization, we expressed wild type SAMHD1, SAMHD1_K11A_, or an unrelated control protein (mCherry) in U937 cells. We then differentiated the cells with PMA for 48 h and challenged with a VSV-G pseudotyped HIV-1 GFP encoding viral vector. Infected cells were enumerated by flow cytometry at 48 h. Expression of either wild type or SAMHD1_K11A_ resulted in similar decreases in infection (Figure 5A) [17,18]. Western blot analysis confirmed comparable levels of expression of the wild type and K11A mutant proteins and the lack of endogenous SAMHD1 expression in U937 cells transduced with the mCherry control (Figure 5B). The SAMHD1 core domain forms oligomers and allosteric sites are found at the interfaces of the monomers, suggesting that enzymatic function may require dimerization or oligomerization [4]. We therefore used GST pull down experiments to establish that wild type GST tagged SAMHD1 interacts with HA-tagged wild type and SAMHD1_K11A_ with similar efficiencies, suggesting that the K11A mutation does not interfere with SAMHD1 multimerization (Additional file 1: Figure S7).

To extend our studies to a more relevant cell type in which SAMHD1 is naturally expressed, we investigated cytoplasmic SAMHD1 sensitivity to Vpx_MAC_ in human MDM. Cells were isolated from 2 independent donors, transduced with lentiviral vectors encoding HA-tagged wild type or SAMHD1_K11A_, or E2-Crimson as a control, in the presence of VLPs with or without Vpx_MAC_. Three days post transduction, the cells were infected with a HIV-1 GFP reporter vector and analyzed by flow cytometry. At the time of challenge, parallel samples were subjected to western blot or immunofluorescence analysis.

Since the presence of Vpx_MAC_ renders MDM more susceptible to lentiviral transduction, we first compared transduction efficiencies in the presence or absence of Vpx_MAC_. We observed 10% of cells expressing E2-Crimson in the absence of Vpx_MAC_ and almost 100% in the presence of Vpx_MAC_, confirming the functionality of Vpx_MAC_ (Additional file 1: Figure S8A). In parallel samples we investigated the levels of ectopic wild type or SAMHD1_K11A_, as well as the endogenous protein. Similar levels of HA-SAMHD1 and HA-SAMHD1_K11A_ were detected in the absence of Vpx_MAC_ (Figure 5C, lane 3 and 5), suggesting comparable transduction efficiencies for both lentiviral vector stocks (Figure 5C and Additional file 1: Figure S8B, C). In the presence of VLPs containing Vpx_MAC_, the level of HA-SAMHD1 was slightly increased (Figure 5C and Additional file 1: Figure S8B, C, lane 3 and 4), whereas the level of HA-SAMHD1_K11A_ was substantially increased (Figure 5C and Additional file 1: Figure S8B, C, lane 5 and 6). We conclude that cytoplasmic SAMHD1 is less sensitive to Vpx_MAC_ initiated degradation in primary MDM than its nuclear localized counterpart. We also investigated the antiviral phenotypes of these cultures and observed a ~2-fold reduction in HIV-1 GFP reporter vector infectivity for HA-SAMHD1, but a substantially stronger reduction of ~5-10-fold in HA-SAMHD1_K11A_ expressing cells (Figure 5D and Additional file 1: Figure S8D, E).

**Figure 6:**
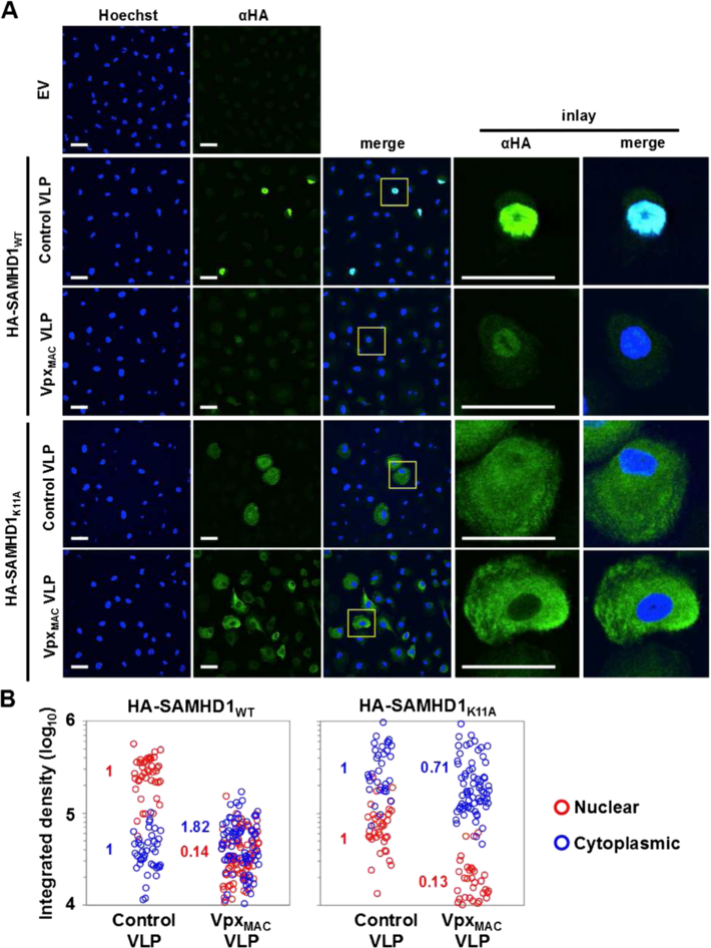
**The nuclear fraction of SAMHD1**_**K11A **_**in MDM is sensitive to Vpx**_**MAC **_**induced degradation. A)** Monocyte-derived macrophages were transduced with lentiviral vectors encoding HA-tagged SAMHD1_WT_ or SAMHD1_K11A_ in the presence of virus like particles (VLPs) delivering Vpx_MAC_ or control VLPs and cells were fixed for HA-immunostaining 72 h later. Scale bars, 30 μm. Shown are representative pictures from one of four independent donors. **B)** The integrated MFI densities were calculated on MDM expressing HA-SAMHD1_WT_ or HA-SAMHD1_K11A_ for nuclear and cytoplasmic areas using ImageJ software. Numbers within the graphs show fold changes of the average integrated densities normalized to control VLP treated cells.

Next we investigated the expression of HA-SAMHD1 and HA-SAMHD1_K11A_ in MDM by anti-HA-immunofluorescence microscopy. In the absence of VLPs or when treated with control VLPs lacking Vpx_MAC_, MDM were transduced with similar efficiencies resulting in 2-10% of cells expressing HA-SAMHD1 proteins (Additional file 1: Figure S9). In the presence of Vpx_MAC_ VLPs we observed a substantial increase in the number of transduced cells expressing HA-SAMHD1_WT_ as well as HA-SAMHD1_K11A_ (Additional file 1: Figure S9). However, the integrated density of nuclear localized HA-SAMHD1_WT_ protein decreased substantially, whereas the cytoplasmic HA-SAMHD1_K11A_ was unaffected (Figure 6 and Additional file 1: Figure S10A). Of note, in untreated MDM or MDM treated with control VLPs, we observed a noticeable amount (~20%) of total HA-SAMHD1_K11A_ protein in the nucleus (Figure 6 and Additional file 1: Figure S10A), with the integrated density of this nuclear fraction but not the cytoplasmic fraction decreasing in the presence of Vpx_MAC_ VLPs (Figure 6 and Additional file 1: Figure S10A), suggesting that some HA-SAMHD1_K11A_ can be imported into the nucleus under certain conditions and acquires sensitivity for Vpx_MAC_ mediated degradation when it does so.

Our results indicate that cytoplasmic SAMHD1_K11A_ can reduce HIV-1 infection in primary MDM and is substantially less sensitive to Vpx_MAC_ mediated suppression relative to nuclear localised SAMHD1. They also imply that SAMHD1_K11A_ can be imported into the nucleus in MDM either by cooperative nuclear import in complex with endogenous SAMHD1 [29], or by alternative nuclear import pathways that are independent of the N-terminal NLS [28].

### Vpx_MAC_ induces ubiquitination of nuclear and cytoplasmic SAMHD1

SAMHD1_K11A_ has reduced sensitivity to Vpx_MAC_ stimulated degradation, but the underlying mechanism has not been defined. The CUL4A/DDB1/DCAF1 ligase complex is found in the nucleus and we speculated that the insensitivity of SAMHD1_K11A_ may be due to the absence or unavailability of the relevant ubiquitination machinery in the cytoplasm.

**Figure 7:**
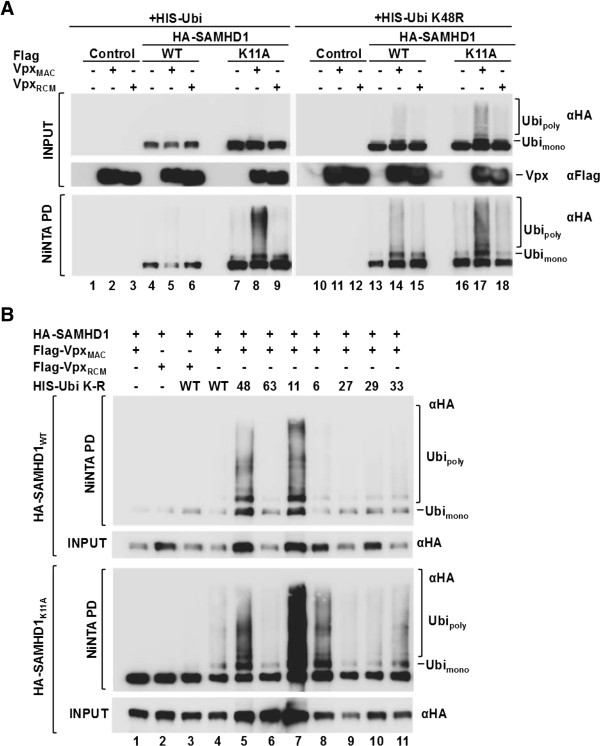
**SAMHD1**_**K11A **_**is ubiquitinated in the presence of Vpx**_**MAC**_**. A)** 293 T cells were co-transfected with plasmids encoding HA-tagged wild type or K11A mutant SAMHD1 along with plasmids expressing Vpx_MAC_ or Vpx_RCM_ and plasmids encoding either HIS-tagged wild type ubiquitin (HIS-Ubi) or K48R mutant ubiquitin (HIS-UbiK48R) for 24 h before cells were lysed and subjected to NiNTA pull down. Cell lysates (Input) or pulled down samples (NiNTA PD) were subjected to SDS-PAGE using 10% gels and western blotting using an anti-HA antibody. Mono- and polyubiquitinated SAMHD1 are indicated. **B)** Similar experiment as shown in A) but using ubiquitin mutants HIS-UbiK6R, K11R, K27R, K29R, K33R, K48R or K63R.

293 T cells were transfected with plasmids encoding wild type or K11A SAMHD1 in the presence or absence of Vpx_MAC_ or Vpx_RCM_ together with hexahistidine-tagged wild type or K48R mutant ubiquitin (Ubi) and subjected to NiNTA pull down. In the presence of wild type HIS-Ubi and Vpx_MAC_, but not Vpx_RCM_, we observed a faint accumulation of slower migrating bands (Figure 7 A, NiNTA PD lane 5 vs. lanes 4 and 6), indicative of polyubiquitinated SAMHD1. These higher molecular mass bands, as well as a band corresponding to monoubiquitinated SAMHD1, accumulated to a greater extent in the presence of the dominant negative ubiquitin mutant UbiK48R (Figure 7A, lane 14) suggesting that K48 linked polyubiquitin chains target wild type SAMHD1 for degradation [35,36]. Intriguingly, when we performed the same experiment with cytoplasmic SAMHD1_K11A_ and wild type HIS-ubiquitin, we observed a substantial accumulation of higher molecular mass bands in the presence of Vpx_MAC_ (Figure 7A, lane 8), suggesting that cytoplasmic SAMHD1 is a good target for Vpx_MAC_ mediated ubiquitination but that the polyubiquitinated product may be less efficiently deubiquitinated and degraded. Higher molecular mass bands were also abundant for SAMHD1_K11A_ in the presence of K48R mutant ubiquitin (Figure 7A, lane 17), indicative of inefficient deubiquitination. In the presence of UbiK48R levels of polyubiquitinated SAMHD1_WT_ chains did not reach the levels observed for SAMHD1_K11A_ (Figure 7A, lane 14 vs. 17), suggesting that additional ubiquitin linkages may be involved in SAMHD1 degradation.

To address the incorporation of other ubiquitin linkages in polyubiquitinated chains we co-transfected HA-SAMHD1_WT_ or HA-SAMHD1_K11A_ expressing plasmids with Flag-Vpx_MAC_ or Flag-Vpx_RCM_ and HIS-tagged wild type ubiquitin or ubiquitin lysine to arginine mutants UbiK6R, UbiK11R, UbiK27R, UbiK29R, UbiK33R, UbiK48R or UbiK63R and performed NiNTA pull down experiments as before. Interestingly, in addition to the enhanced accumulation of chains in the presence of UbiK48R, we also observed higher levels of polyubiquitin chains in the presence of UbiK11R (Figure 7B, lane 5 vs. 7), suggesting that K11 linked ubiquitin chains may also form during Vpx_MAC_ induced degradation of SAMHD1_WT_. We also observed a very strong accumulation of higher molecular mass bands with SAMHD1_K11A_ in the presence of UbiK11R and a moderate increase in the presence of UbiK6R, indicative of perturbed deubiquitination in the presence of either of these mutants (Figure 7B, lane 7 and 8) as well as with K48R.

## Discussion

Here we have investigated the nuclear localization requirements for SAMHD1 and the consequences of its disruption. In cells from healthy human donors, SAMHD1 appears to be localized exclusively to the nucleus, whereas in cells of Aicardi-Goutières syndrome (AGS) patients with mutations in *SAMHD1* cytoplasmic accumulation of SAMHD1 has been observed [15,16]. We have identified a classical/basic NLS in the N-terminus of SAMHD1 (^11^KRPR^14^) and have shown that substitutions of critical amino acids in this motif result in cytoplasmic accumulation in MDM (Figure 1E). Consistent with recently published studies, NLS function can be transferred to a heterologous protein (Figure 1C) [17] and the SAMHD1 NLS can be substituted by a heterologous NLS (Figure 1D). Importantly, ~80-95% of the SAMHD1_K11A_ NLS-mutant protein was localized to the cytoplasm in both HeLa cells as well as MDM, suggesting that the N-terminal NLS constitutes the major signal for nuclear import in cell lines as well as primary cells.

Systematic analysis of human KPNAs identified KPNA2 as the principal nuclear import receptor binding to the N-terminal SAMHD1 NLS (Figure 2), though a minor, possibly redundant, role for KPNA6 could not be ruled out (Additional file 1: Figure S3). We further confirmed utilization of the classical nuclear import pathway by demonstrating the importance of KPNB1 using RNAi (Figure 2). These data therefore indicate that human SAMHD1 nuclear import is primarily mediated through the interaction of KPNA2 with the N-terminal ^11^KRPR^14^ motif and engagement of KPNB1. Of note, two unrelated proteins, NBS1 and Oct4, contain almost identical NLSs only differing in the third amino acid position, ^465^KRER^468^ and ^61^KRKR^64^, respectively and both have been demonstrated to bind and require KPNA2 for nuclear import [37-40]. We did not observe a complete block for SAMHD1 nuclear import in cells treated with KPNA2 or KPNB1 specific shRNAs, which may be explained by cytotoxicity when KPNA2 or KPNB1 levels are largely ablated, or may reflect a degree of redundancy between nuclear import receptors. More specifically, we observed that KPNA1 interacts with both SAMHD1_WT_ as well as SAMHD1_K11A_ suggesting that SAMHD1 contains a KPNA1 binding site that is discrete from the N-terminal NLS. Although karyopherin α proteins other than KPNA2 may contribute to SAMHD1 nuclear import, it is likely that any such effects are of minor importance given that mutation of the N-terminal NLS is sufficient to re-distribute 80-95% of total SAMHD1 to the cytoplasm. However, we found that in MDM SAMHD1_K11A_ was not completely excluded from the nucleus (Figure 1). The modest nuclear accumulation of SAMHD1_K11A_ in MDM may be due to such activities, or assisted import as a complex with endogenous SAMHD1, as has been suggested for other proteins [29] and is supported by the data of Guo et al. [28].

Surprisingly, substitution of the NLS in human SAMHD1 with corresponding sequences from certain species caused cytoplasmic accumulation, suggesting that the NLS is not highly conserved between different species. Interestingly, African green monkeys contain two different SAMHD1 NLS sequences [11] [KRLH (*Chlorocebus tantalus*) and KRLR (*Chlorocebus pygerythrus*)] leading to cytoplasmic or nuclear localization of SAMHD1, respectively (Additional file 1: Figure S6). The physiological relevance of these differences is not yet known but may help illuminate the impact of SAMHD1 mislocalization on AGS [16]. Importantly, we investigated the antiviral activity of cytoplasmic and nuclear SAMHD1 in MDM and confirmed that each form inhibited HIV-1 infection to a comparable degree (Figure 5) [17,18]. This suggests that if mislocalization of mutated SAMHD1 contributes to the development of AGS, then SAMHD1′s HIV-1 restriction activity may be mechanistically distinct from its physiologic function in human cells.

Cytoplasmic SAMHD1 is less sensitive to Vpx_MAC_ mediated degradation in MDM (Figure 6), in keeping with what has been shown in cell lines [17,18]. The mechanistic basis for the reduced sensitivity of cytoplasmic SAMHD1 to Vpx_MAC_ induced degradation can not be explained by reduced Vpx_MAC_ binding (Figure 3) [18]. We therefore examined whether cytoplasmic SAMHD1 is ubiquitinated in response to Vpx_MAC_. The degradation of nuclear (wild type) SAMHD1 in the presence of Vpx_MAC_ was partially prevented by the presence of dominant negative ubiquitin mutants K48R or K11R, suggesting that K48- as well as K11-linked polyubiquitination may precede SAMHD1 proteasomal degradation (Figure 7). Cytoplasmic SAMHD1_K11A_ was also targeted for ubiquitination by Vpx_MAC_ (Figure 7), and our data invoke (at least) two plausible explanations for the reduced sensitivity of cytoplasmic SAMHD1 to Vpx_MAC_ induced degradation: 1) wild type SAMHD1 may be degraded using proteasomal machinery components such as deubiquitinating enzymes (DUBs) that are either confined to the nucleus, or only active in the nucleus; and/or 2) ubiquitin chains that are appended to cytoplasmic SAMHD1 may differ in the linkage patterns, leading to resistance to degradation. Using ubiquitin lysine to arginine mutants we observed differences in the polyubiquitination profiles for wild type and K11A SAMHD1, specifically with UbiK11R and UbiK6R, leading to increases in polyubiquitinated SAMHD1_K11A_ compared to SAMHD1_WT_ (Figure 7B, lanes 7 and 8). Importantly, DUBs that specifically modulate ubiquitination in the nucleus have been described before [41,42] and future investigations of SAMHD1 will explore these possible scenarios with a view to providing insight into the nucleocytoplasmic partitioning of elements of the ubiquitin/proteasome system.

## Conclusions

Our work extends published observations on SAMHD1 nuclear localization to a natural cell type for HIV-1 infection, identifies KPNA2/KPNB1 as cellular factors important for SAMHD1 nuclear import, and indicates that components of the nuclear proteasomal degradation machinery are required for Vpx induced SAMHD1 degradation.

## Methods

### Cell culture and stable cell lines

293 T, HEK-Blue™ (Invivogen) and Hela cells were grown in Dulbecco’s modified Eagle medium (DMEM); U937, THP-1 and primary monocyte-derived macrophages (MDM) were grown in RPMI 1640 Glutamax + HEPES medium supplemented with 10% fetal bovine serum (FBS) (Gibco) and penicillin-streptomycin. MDM were obtained from peripheral blood mononuclear cells (PBMCs) of healthy volunteer donors. Briefly, PBMCs were isolated using Lymphoprep (Axis-Shield) and monocytes were obtained by positive selection using CD14 specific MicroBeads (Miltenyi Biotec). After 3 h in serum-free RPMI 1640 medium, monocytes were differentiated into macrophages by culture for 4 to 10 days in growth media supplemented with 100 ng/ml granulocyte-macrophage colony stimulating factor (GM-CSF) (R&D Systems).

### Plasmids and shRNAs

U937 cells were transduced with the HIV-1 based lentiviral vector, pCSCW, where the human SAMHD1 cDNA had replaced the mCherry open reading frame to express untagged wild type or K11A mutant SAMHD1, pCSwtW and pCSk11aW, respectively. For ectopic expression in monocyte-derived macrophages (MDM) a HA-tag coding sequence was inserted 5′ of the SAMHD1 open reading frame generating pCSHAwtW and pCSHAk11aW. To generate a control that was suitable for measuring MDM transduction efficiency by flow cytometry the E2-Crimson cDNA was inserted to replace mCherry, generating pCSE2W. For glutathione S-transferase (GST) pull-down experiments, GST-tagged SAMHD1 was expressed from pCAGGS [43]. Haemagglutinin (HA)-tagged wild type or K11A mutant SAMHD1, Turbo-RFP (tRFP) or MKRPR-tRFP, as well as human karyopherins, were expressed from pLNCX2 based MLV vectors [44]. Yellow fluorescence protein (YFP)-tagged wild type or mutant SAMHD1 proteins were expressed from the puromycin selectable MIGR-I based MLV vector pCMS28YFP [43]. HIV-1 or MoMLV based GagPol expression plasmids and VSV-G encoding plasmid pMD.G have been described [44]. Short hairpin RNAs targeting human karyopherins (Additional file 1: Table S1) were designed using the Clontech shRNA software (http://www.clontech.com/GB/Support) inserted into pSIREN.Retro-Q (Clontech) and expressed from the human U6 promoter as described before [45]. Flag-tagged Vpx proteins from HIV-2_ROD_ or SIVs from red-capped mangabey (RCM) or rhesus macaque (MAC) were expressed from HIV-1 based lentiviral vectors as described [46]. Hexahistidine (6HIS)-tagged wild type or K6R, K11R, K27R, K29R, K33R, K48R and K63R ubiquitin was expressed from lentiviral vector pHRSIN-6HIS-Ubi-GFP [47].

### Viral vector production

293 T cells were transfected in 10 cm plates at a confluence of ~80% with 3 μg viral vector plasmid, 2 μg GagPol encoding plasmid (p8.91 for HIV-1 based vectors, and pCMVi [44] for MLV based vectors) and 2 μg pMD.G using 4 μg polyethylenimine (PEI) per μg DNA in 1 ml of OptiMEM (Gibco) per 10 cm plate. The medium was changed 24 h post transfection, the virus was harvested at 48 h and 72 h post transfection, passed through a 0.45 μm filter and the collections were pooled. Depending on the experiment, viral vector supernatants were subjected to purification through a sucrose cushion [46].

### Antibodies

Antibodies used for western blots were: mouse monoclonal SAMHD1 antibody 1 F9 at dilution 1/3000 (Abcam, ab117908), mouse monoclonal α-tubulin antibody DM1A at dilution 1/3000 (Sigma Aldrich, T9026), mouse monoclonal GST antibody at dilution 1/10000 [43], mouse monoclonal KPNB1 antibody 3E9 (Abcam, ab2811), rabbit polyclonal Hsp90 antibody H-114 at dilution 1/3000 (Santa Cruz Biotechnology, sc-7947), HRP-linked mouse monoclonal Flag antibody M2 at dilution 1/3000 (Sigma Aldrich, A8592) and HRP-linked rat monoclonal HA antibody 3 F10 at dilution 1/5000 (Roche Applied Science, 12013819001). For immunofluorescence staining mouse monoclonal HA antibody 12CA5 was used at dilution 1/250. For immunoprecipitation we used HA antibody 12CA5 at a dilution of and GFP antibody (Roche Applied Science, 11814460001) at a dilution of.

### Immunofluorescence & microscopy

HeLa cells were stably transduced with LNCX2 based MLV vectors expressing HA-tagged wild type or K11A mutant SAMHD1 and selected with 100 μg/ml G418 for two weeks. Stable drug selected cells were seeded at ~50% density on coverslips in 24-well plates and fixed with 4% paraformaldehyde (PFA) 24 h after plating. Cells were permeabilised with 0.1% Triton X-100 washed twice in PBS and blocked in NGB buffer (50 mM NH_4_Cl, 1% goat serum, 1% bovine serum albumin) for 1 h, as described before [48]. The cells were incubated with primary antibody diluted 1/250 in NGB buffer at room temperature for 1 h, washed three times in PBS and incubated for 1 h with species-specific Alexa Fluor 488- or 594-conjugated secondary antibody (Molecular Probes) diluted 1/500 in NGB buffer. Cells were washed three times in PBS and incubated with 4′,6′-diamidino-2-phenylindole (DAPI) dilactate (0.1 μg/ml, Molecular Probes) or Hoechst 33258 (1 μg/ml, Sigma Aldrich) for nuclei staining, washed again and mounted on glass slides using Mowiol (Calbiochem). For autofluorescence imaging, 293 T cells were stably transduced with pCMS28YFP_WT_ or pCMS28YFP_K11A_ encoding N-terminal YFP-tagged wild type or K11A mutant SAMHD1, respectively, and selected with 1 μg/ml puromycin for one week. Stably transduced cells were seeded on coverslips in 24-well plates, fixed using 4% paraformaldehyde and incubated with DAPI or Hoechst before mounting. For karyopherin knock down experiments, 293 T cells expressing wild type YFP-tagged SAMHD1 were seeded on coverslips as before and transduced once with MLV SIREN-RetroQ vector expressing shRNAs targeting the different karyopherins for 48 h before the cells were fixed and mounted as before. Laser scanning confocal microscopy was performed on a DM IRE2 microscope (Leica), and images were processed and analysed using LCS (Leica), ImageJ (NIH) and Photoshop (Adobe) software packages.

### Image quantification

HeLa cells or MDM transduced with HA empty vector (EV) control were stained in parallel to SAMHD1_WT_ and SAMHD1_K11A_ transduced cells with anti-HA antibody as described above. From HA-EV control cells average MFI background stainings for the nuclear and cytoplasmic areas were calculated per pixel using ImageJ by defining an area of interest around the nucleus as defined by DAPI staining and around the whole cell (cytoplasmic MFI was derived by subtracting nuclear MFI from whole cell MFI). For HA-SAMHD1_WT_ as well as HA-SAMHD1_K11A_ expressing cells the pixel number of nuclear and whole cell areas was multiplied with the average background MFI per pixel for each area (as derived from the HA-EV control) and this value was subtracted from the integrated density (MFI × area) of the nuclear or cytoplasmic area for HA-SAMHD1 expressing cells using ImageJ. Percentage of nuclear SAMHD1 was calculated as the ratio of the integrated densities of nuclear SAMHD1 to whole cell associated SAMHD1.

### GST-pull downs

293 T cells were seeded at 6 10^5^ per well in 6-well plates and transfected the next day using PEI with 2 μg of each GST vector and HA–SAMHD1, HA-KPNA or Flag-Vpx vectors. The media was replaced 24 h post transfection. Cells were washed once in 1 ml PBS and then harvested in 1 ml lysis buffer (50 mM Tris-HCl, pH 7.4; 150 mM NaCl; 5 mM EDTA; 5% glycerol; 1% Triton X-100; Complete Protease inhibitor (Roche Diagnostics)) 48 h post transfection. Lysed cells were incubated on a rotating wheel at 4°C for 20 min, then clarified using a bench top centrifuge at 13,000 × g at 4°C for 15 min. For input analysis 50 μl were removed, mixed with 50 μl 2× Laemmli buffer and boiled. The remaining cell lysate was incubated with 25 μl glutathione-sepharose beads (50% solution in wash buffer) for 3 h at 4°C on a rotating wheel. Beads were washed three times in wash buffer (50 mM Tris-Hcl, pH 7.4; 150 mM NaCl; 5 mM EDTA; 5% glycerol; 0.1% Triton X-100; Complete Protease Inhibitor (Roche Diagnostics)) and proteins were eluted by boiling in 50 μl 2× Laemmli buffer. Protein samples were analysed by SDS-PAGE and western blotting.

### SAMHD1 degradation assay

293 T cells were seeded at 1 × 10^5^ per well in 24-well plates and co-transfected with 100 ng pCMS28YFPSAMHD1_WT_ or pCMS28YFPSAMHD1_K11A_ plasmids and 100 to 1000 ng Flag-tagged Vpx_MAC_, Vpx_RCM_ or Vpx_HIV-2ROD_ encoding plasmid. The percentage of YFP expressing cells, as well as the MFI were measured 24 h post transfection by flow cytometry using a FACS Calibur machine and CELLQuest software (BD). For SAMHD1 degradation assays in THP-1 we plated 2 10^5^ × YFP-SAMHD1_WT_ or YFP-SAMHD1_K11A_ expressing cells into 48-well plate dishes and added sucrose-purified and concentrated Vpx_MAC_ containing or Vpx-deficient control VLPs corresponding to 10 ng reverse transcriptase (RT) or left cells untreated. After indicated time points cells were harvested and cell lysates were subjected to western blot analysis.

### S^35^ pulse chase labeling to measure SAMHD1 stability

293 T cells were seeded at 5 × 10^5^ per well in 6-well plates and transfected the next day with 1 μg of YFP-SAMHD1_WT_, HA-SAMHD1_WT_ or HA-SAMHD1_K11A_ expressing plasmids using 4 μg polyethylenimine (PEI) per μg DNA in 0.5 ml of OptiMEM (Gibco) per well. Twenty-four hours later cells were washed twice in cysteine/methionine depleted DMEM (Gibco) and incubated for 20 min at 37°C in depletion media. The media was aspirated and replaced by pulse labeling media (depletion media containing 0.25 mCi/ ml S^35^ labeled cysteine/methionine; S^35^-Cys/Met EXPRESS, Perkin Elmer) for 10 min before trypsinising cells. Cells were washed three times in 10 ml DMEM and replated in DMEM into 6-well plate dishes. The 0 h time point was collected at the time of cell plating and subsequent time points were collected as indicated. Cells were lysed in 500 μl lysis buffer (50 mM Tris-HCL (pH7.6), 150 mM NaCl, 1% Triton X-100) and incubated on ice for 10 minutes. Cell lysates were pelleted at 1000 g for 10 minutes to remove insoluble material. Lysate supernatants were incubated with antibody (anti HA or anti GFP) -conjugated magnetic protein G beads (GE Healthcare) for 2 h at 4°C while rocking. Beads were washed three times in lysis buffer, eluted with Laemmli protein loading buffer, boiled and separated by 10% SDS PAGE. Gels were dried and exposed on a phosphor image screen over several days before development on a Typhoon Trio phosphorimager (GE Healthcare).

### Ectopic gene expression in human monocyte derived macrophages

MDM were isolated and differentiated as described above [49], seeded in 48-well plates at 2 × 10^5^ or 24-well plates for immunofluorescence at 5 × 10^6^ cells per well, respectively. For ectopic gene expression, macrophages were treated with purified SIV_MAC_ Vpx VLPs corresponding to 10 to 20 ng reverse transcriptase (RT) as measured by the colorimetric reverse transcriptase assay (Roche) and CSE2W, CSHAwtW or CSHAk11aW derived HIV-1 vector supernatants. Three days after transduction, the supernatants were harvested and tested for the presence of type I IFN using HEK-Blue™ cells and the transduced macrophages were challenged with at least four different doses of HIV-1 GFP reporter vector CSGW derived viral supernatants. At the time of infection with the GFP reporter virus parallel samples were harvested for western blot or fixed for immunofluorescence analysis. The infectivity of the GFP reporter virus was determined two days after infection by flow cytometry. Transduction efficiency of the MDM was evaluated by measuring percent of E2-Crimson positive cells using flow cytometry 72 h after transduction.

### Pull down of hexahistidine-tagged proteins

Pull down of hexahistidine-ubiquitinated proteins in mammalian cells has been described [50]. Briefly, 293 T cells were seeded at 6 × 10^5^ cells per well in 6-well plates and transfected using PEI with 1 μg pHRSINGFP-6HIS -Ubi_WT_ or -Ubi_K-R_ mutant plasmids, MLV based vector encoding HA-tagged wild type or K11A SAMHD1 or empty vector control, as well as Flag-tagged Vpx_MAC_ or Vpx_RCM_. The medium was replaced 24 h post transfection. Cells were washed once 48 h post transfection in 1 ml PBS and then harvested in 1 ml ice cold PBS. An aliquot was reserved as the input sample, mixed with 2× Laemmli buffer and boiled. The remaining cells were resuspended in 1 ml lysis buffer (6 M guanidine-HCl, 0.1 M Na_2_HPO_4_/NaH_2_PO_4_, 10 mM imidazole, pH 8.0) and subjected to 10 s sonication using a small tip at level 2 before adding 50 μl of equilibrated (50%) NiNTA-agarose (Qiagen). The lysates were incubated rotating at room temperature for 3 h. The NiNTA-agarose was washed two times in 1 ml lysis buffer, two times in buffer L/TI (1 volume lysis buffer + 3 volumes buffer TI [25 mM Tris/HCl, 20 mM imidazole, pH 6.8]) and one time in buffer TI. Bound proteins were eluted by boiling in 100 μl 2× Laemmli buffer with 300 mM imidazole and separated by SDS-PAGE.

### SAMHD1 sequence accession numbers

SAMHD1 sequences were derived from GenBank or Ensembl databases and had following accession numbers: *Homo sapiens* [GenBank: NM_015474.3], *Pan troglodytes* [GenBank: NM_001280510.1]*, Pongo abelii* [GenBank: XM_002830274.2]*, Gorilla gorilla* [GenBank: NM_001279619.1]*, Hylobates agilis* [GenBank: JQ231127.1]*, Macaca mulatta* [GenBank: NM_001271642.1], *Nomascus leucogenys* [GenBank: JQ231129.1], *Callicebus molloch* [GenBank: JQ231152.1], *Nasalis larvatus* [GenBank: JQ231144.1]*, Lagothrix lagotricha* [GenBank: JQ231150.1], *Callithrix jacchus* [GenBank: JN936906.1], *Alenopithecus nigroviridis* [GenBank: JQ231142.1], *Erythrocebus patas* [GenBank: JQ231138.1], *Chlorocebus tantalus* [GenBank: JQ231136.1], *Chlorocebus pygerythrus* [GenBank: JQ231137.1], *Aotus trivirgatus* [GenBank: JQ231148.1], *Cercopithecus neglectus* [GenBank: JQ231141.1]*, Pteropus vampyrus* [Ensembl: ENSPVAT00000010770]*, Myotis lucifugus* [Ensembl: ENSMLUT00000002224]*, Echinops telfairi* [Ensembl: ENSETET00000002844], *Monodelphis domestica* [GenBank: XM_001381548.2]*, Erinaceus europaeus* [Ensembl: ENSEEUT00000004266]*, Anolis carolinensis* [GenBank: XM_003220542.1]*, Gallus gallus* [GenBank: NM_001030845.1], *Rattus norvegicus* [GenBank: NM_001191743.1], *Mus musculus* [GenBank: NM_018851.3], *Oryctolagus cuniculus* [Ensembl: ENSOCUT00000007611]*, Danio rerio* [GenBank: NM_001159933.1], *Felis catus* [Ensembl: ENSFCAT00000012967], *Canis familiaris* [Ensembl: ENSCAFT00000013745], *Ailuropoda melanoleuca* [Ensembl: ENSAMET00000013697], *Tursiops truncates* [Ensembl: ENSTTRT00000001930], *Ochotona princeps* [Ensembl: ENSOPRT00000012314], *Dipodomys ordii* [Ensembl: ENSDORT00000012349], *Otolemur garnettii* [Ensembl: ENSOGAT00000000909], *Microcebus murinus* [Ensembl: ENSMICT00000002449], *Equus caballus* [Ensembl: ENSECAT00000009592], *Bos taurus* [Ensembl: ENSBTAT00000043682], *Sorex araneus* [Ensembl: ENSSART00000011255], *Macropus eugenii* [Ensembl: ENSMEUT00000000939].

## Competing interests

The authors declare that they have no competing interests.

## Authors’ contributions

TS and MHM designed the study and wrote the manuscript. TS, DP and LA carried out the experiments. CG provided reagents. All authors read and approved the final manuscript.

## Supplementary Material

Additional file 1 Contains supplementary Table S1 and supplementary figures S1-S10 with supporting data to the main figures. Click here for file

## References

[B1] Sayah DM Sokolskaja E Berthoux L Luban J Cyclophilin A retrotransposition into TRIM5 explains owl monkey resistance to HIV-1 Nature 2004 430 569 573 10.1038/nature02777 15243629

[B2] Stremlau M Owens CM Perron MJ Kiessling M Autissier P Sodroski J The cytoplasmic body component TRIM5alpha restricts HIV-1 infection in old world monkeys Nature 2004 427 848 853 10.1038/nature02343 14985764

[B3] Sheehy AM Gaddis NC Malim MH The antiretroviral enzyme APOBEC3G is degraded by the proteasome in response to HIV-1 Vif Nat Med 2003 9 1404 1407 10.1038/nm945 14528300

[B4] Goldstone DC Ennis-Adeniran V Hedden JJ Groom HC Rice GI Christodoulou E Walker PA Kelly G Haire LF Yap MW de Carvalho LP Stoye JP Crow YJ Taylor IA Webb M HIV-1 restriction factor SAMHD1 is a deoxynucleoside triphosphate triphosphohydrolase Nature 2011 480 379 382 10.1038/nature10623 22056990

[B5] Hrecka K Hao C Gierszewska M Swanson SK Kesik-Brodacka M Srivastava S Florens L Washburn MP Skowronski J Vpx relieves inhibition of HIV-1 infection of macrophages mediated by the SAMHD1 protein Nature 2011 474 658 661 10.1038/nature10195 21720370 PMC3179858

[B6] Laguette N Sobhian B Casartelli N Ringeard M Chable-Bessia C Segeral E Yatim A Emiliani S Schwartz O Benkirane M SAMHD1 is the dendritic- and myeloid-cell-specific HIV-1 restriction factor counteracted by Vpx Nature 2011 474 654 657 10.1038/nature10117 21613998 PMC3595993

[B7] Powell RD Holland PJ Hollis T Perrino FW Aicardi-goutieres syndrome gene and HIV-1 restriction factor SAMHD1 is a dGTP-regulated deoxynucleotide triphosphohydrolase The Journal of biological chemistry 2011 286 43596 43600 10.1074/jbc.C111.317628 22069334 PMC3243528

[B8] Baldauf HM Pan X Erikson E Schmidt S Daddacha W Burggraf M Schenkova K Ambiel I Wabnitz G Gramberg T Panitz S Flory E Landau NR Sertel S Rutsch F Lasitschka F Kim B Konig R Fackler OT Keppler OT SAMHD1 restricts HIV-1 infection in resting CD4(+) T cells Nature medicine 2012 18 1682 1689 10.1038/nm.2964 22972397 PMC3828732

[B9] Descours B Cribier A Chable-Bessia C Ayinde D Rice G Crow Y Yatim A Schawartz O Laguette N Benkirane M SAMHD1 restricts HIV-1 reverse transcription in quiescent CD4+ T-cells Retrovirology 2012 9 87 10.1186/1742-4690-9-87 23092122 PMC3494655

[B10] Laguette N Rahm N Sobhian B Chable-Bessia C Munch J Snoeck J Sauter D Switzer WM Heneine W Kirchhoff F Delsuc F Telenti A Benkirane M Evolutionary and functional analyses of the interaction between the myeloid restriction factor SAMHD1 and the lentiviral Vpx protein Cell host & microbe 2012 11 205 217 10.1016/j.chom.2012.01.007 22305291 PMC3595996

[B11] Lim ES Fregoso OI McCoy CO Matsen FA Malik HS Emerman M The ability of primate lentiviruses to degrade the monocyte restriction factor SAMHD1 preceded the birth of the viral accessory protein Vpx Cell host & microbe 2012 11 194 204 10.1016/j.chom.2012.01.004 22284954 PMC3288607

[B12] Goujon C Riviere L Jarrosson-Wuilleme L Bernaud J Rigal D Darlix JL Cimarelli A SIVSM/HIV-2 Vpx proteins promote retroviral escape from a proteasome-dependent restriction pathway present in human dendritic cells Retrovirology 2007 4 2 10.1186/1742-4690-4-2 17212817 PMC1779362

[B13] Manel N Hogstad B Wang Y Levy DE Unutmaz D Littman DR A cryptic sensor for HIV-1 activates antiviral innate immunity in dendritic cells Nature 2010 467 214 217 10.1038/nature09337 20829794 PMC3051279

[B14] Crow YJ Aicardi-Goutières syndrome GeneReviews 2005 [Internet, Updated 2014 Mar 13]

[B15] Rice GI Bond J Asipu A Brunette RL Manfield IW Carr IM Fuller JC Jackson RM Lamb T Briggs TA Ali M Gornall H Couthard LR Aeby A Attard-Montalto SP Bertini E Bodemer C Brockmann K Brueton LA Corry PC Desguerre I Fazzi E Cazorla AG Gener B Hamel BC Heiberg A Hunter M van der Knaap MS Kumar R Lagae L Mutations involved in aicardi-goutieres syndrome implicate SAMHD1 as regulator of the innate immune response Nat Genet 2009 41 829 832 10.1038/ng.373 19525956 PMC4154505

[B16] Goncalves A Karayel E Rice GI Bennett KL Crow YJ Superti-Furga G Burckstummer T SAMHD1 is a nucleic-acid binding protein that is mislocalized due to aicardi-goutieres syndrome-associated mutations Human mutation 2012 33 1116 1122 10.1002/humu.22087 22461318

[B17] Brandariz-Nunez A Valle-Casuso JC White TE Laguette N Benkirane M Brojatsch J Diaz-Griffero F Role of SAMHD1 nuclear localization in restriction of HIV-1 and SIVmac Retrovirology 2012 9 49 10.1186/1742-4690-9-49 22691373 PMC3410799

[B18] Hofmann H Logue EC Bloch N Daddacha W Polsky SB Schultz ML Kim B Landau NR The Vpx lentiviral accessory protein targets SAMHD1 for degradation in the nucleus Journal of virology 2012 86 12552 12560 10.1128/JVI.01657-12 22973040 PMC3497686

[B19] Wei W Guo H Han X Liu X Zhou X Zhang W Yu XF A novel DCAF1-binding motif required for Vpx-mediated degradation of nuclear SAMHD1 and Vpr-induced G2 arrest Cellular microbiology 2012 14 1745 1756 10.1111/j.1462-5822.2012.01835.x 22776683

[B20] Pemberton LF Blobel G Rosenblum JS Transport routes through the nuclear pore complex Curr Opin Cell Biol 1998 10 392 399 10.1016/S0955-0674(98)80016-1 9640541

[B21] Kelley JB Talley AM Spencer A Gioeli D Paschal BM Karyopherin alpha7 (KPNA7), a divergent member of the importin alpha family of nuclear import receptors BMC Cell Biol 2010 11 63 10.1186/1471-2121-11-63 20701745 PMC2929220

[B22] Kosugi S Hasebe M Matsumura N Takashima H Miyamoto-Sato E Tomita M Yanagawa H Six classes of nuclear localization signals specific to different binding grooves of importin alpha J Biol Chem 2009 284 478 485 10.1074/jbc.M807017200 19001369

[B23] Mason DA Fleming RJ Goldfarb DS Drosophila melanogaster importin alpha1 and alpha3 can replace importin alpha2 during spermatogenesis but not oogenesis Genetics 2002 161 157 170 12019231 10.1093/genetics/161.1.157 PMC1462091

[B24] Geles KG Adam SA Germline and developmental roles of the nuclear transport factor importin alpha3 in C. elegans Development 2001 128 1817 1830 11311162 10.1242/dev.128.10.1817

[B25] Kamikawa Y Yasuhara N Yoneda Y Cell type-specific transcriptional regulation of the gene encoding importin-alpha1 Experimental cell research 2011 317 1970 1978 10.1016/j.yexcr.2011.05.024 21664354

[B26] Yasuhara N Oka M Yoneda Y The role of the nuclear transport system in cell differentiation Seminars in cell & developmental biology 2009 20 590 599 10.1016/j.semcdb.2009.05.003 19465141

[B27] Yasuhara N Takeda E Inoue H Kotera I Yoneda Y Importin alpha/beta-mediated nuclear protein import is regulated in a cell cycle-dependent manner Experimental cell research 2004 297 285 293 10.1016/j.yexcr.2004.03.010 15194443

[B28] Guo H Wei W Wei Z Liu X Evans SL Yang W Wang H Guo Y Zhao K Zhou JY Yu XF Identification of critical regions in human SAMHD1 required for nuclear localization and Vpx-mediated degradation PloS one 2013 8 e66201 10.1371/journal.pone.0066201 23874389 PMC3708934

[B29] Yu JH Lin BY Deng W Broker TR Chow LT Mitogen-activated protein kinases activate the nuclear localization sequence of human papillomavirus type 11 E1 DNA helicase to promote efficient nuclear import Journal of virology 2007 81 5066 5078 10.1128/JVI.02480-06 17344281 PMC1900230

[B30] Gorlich D Kostka S Kraft R Dingwall C Laskey RA Hartmann E Prehn S Two different subunits of importin cooperate to recognize nuclear localization signals and bind them to the nuclear envelope Current biology: CB 1995 5 383 392 10.1016/S0960-9822(95)00079-0 7627554

[B31] Percipalle P Clarkson WD Kent HM Rhodes D Stewart M Molecular interactions between the importin alpha/beta heterodimer and proteins involved in vertebrate nuclear protein import Journal of molecular biology 1997 266 722 732 10.1006/jmbi.1996.0801 9102465

[B32] Sharova N Wu Y Zhu X Stranska R Kaushik R Sharkey M Stevenson M Primate lentiviral Vpx commandeers DDB1 to counteract a macrophage restriction PLoS Pathog 2008 4 e1000057 10.1371/journal.ppat.1000057 18451984 PMC2323106

[B33] Srivastava S Swanson SK Manel N Florens L Washburn MP Skowronski J Lentiviral Vpx accessory factor targets VprBP/DCAF1 substrate adaptor for cullin 4 E3 ubiquitin ligase to enable macrophage infection PLoS pathogens 2008 4 e1000059 10.1371/journal.ppat.1000059 18464893 PMC2330158

[B34] Schwefel D Groom HC Boucherit VC Christodoulou E Walker PA Stoye JP Bishop KN Taylor IA Structural basis of lentiviral subversion of a cellular protein degradation pathway Nature 2014 505 234 238 24336198 10.1038/nature12815 PMC3886899

[B35] Ward CL Omura S Kopito RR Degradation of CFTR by the ubiquitin-proteasome pathway Cell 1995 83 121 127 10.1016/0092-8674(95)90240-6 7553863

[B36] Kolodziejski PJ Musial A Koo JS Eissa NT Ubiquitination of inducible nitric oxide synthase is required for its degradation Proc Natl Acad Sci U S A 2002 99 12315 12320 10.1073/pnas.192345199 12221289 PMC129442

[B37] Pan G Qin B Liu N Scholer HR Pei D Identification of a nuclear localization signal in OCT4 and generation of a dominant negative mutant by its ablation The Journal of biological chemistry 2004 279 37013 37020 10.1074/jbc.M405117200 15218026

[B38] Li X Sun L Jin Y Identification of karyopherin-alpha 2 as an Oct4 associated protein Journal of genetics and genomics = Yi chuan xue bao 2008 35 723 728 10.1016/S1673-8527(08)60227-1 19103427

[B39] Li XL Jia LL Shi MM Li X Li ZH Li HF Wang EH Jia XS Downregulation of KPNA2 in non-small-cell lung cancer is associated with Oct4 expression Journal of translational medicine 2013 11 232 10.1186/1479-5876-11-232 24070213 PMC3849263

[B40] Tseng SF Chang CY Wu KJ Teng SC Importin KPNA2 is required for proper nuclear localization and multiple functions of NBS1 The Journal of biological chemistry 2005 280 39594 39600 10.1074/jbc.M508425200 16188882

[B41] Dirac AM Bernards R The deubiquitinating enzyme USP26 is a regulator of androgen receptor signaling Molecular cancer research: MCR 2010 8 844 854 10.1158/1541-7786.MCR-09-0424 20501646

[B42] Ventii KH Devi NS Friedrich KL Chernova TA Tighiouart M Van Meir EG Wilkinson KD BRCA1-associated protein-1 is a tumor suppressor that requires deubiquitinating activity and nuclear localization Cancer research 2008 68 6953 6962 10.1158/0008-5472.CAN-08-0365 18757409 PMC2736608

[B43] Carlton JG Caballe A Agromayor M Kloc M Martin-Serrano J ESCRT-III governs the aurora B-mediated abscission checkpoint through CHMP4C Science 2012 336 220 225 10.1126/science.1217180 22422861 PMC3998087

[B44] Schaller T Ocwieja KE Rasaiyaah J Price AJ Brady TL Roth SL Hue S Fletcher AJ Lee K KewalRamani VN Noursadeghi M Jenner RG James LC Bushman FD Towers GJ HIV-1 capsid-cyclophilin interactions determine nuclear import pathway, integration targeting and replication efficiency PLoS pathogens 2011 7 e1002439 10.1371/journal.ppat.1002439 22174692 PMC3234246

[B45] Schaller T Hue S Towers GJ An active TRIM5 protein in rabbits indicates a common antiviral ancestor for mammalian TRIM5 proteins Journal of virology 2007 81 11713 11721 10.1128/JVI.01468-07 17728224 PMC2168759

[B46] Goujon C Arfi V Pertel T Luban J Lienard J Rigal D Darlix JL Cimarelli A Characterization of simian immunodeficiency virus SIVSM/human immunodeficiency virus type 2 Vpx function in human myeloid cells Journal of virology 2008 82 12335 12345 10.1128/JVI.01181-08 18829761 PMC2593360

[B47] Boname JM Thomas M Stagg HR Xu P Peng J Lehner PJ Efficient internalization of MHC I requires lysine-11 and lysine-63 mixed linkage polyubiquitin chains Traffic 2010 11 210 220 10.1111/j.1600-0854.2009.01011.x 19948006 PMC3551259

[B48] Phalora PK Sherer NM Wolinsky SM Swanson CM Malim MH HIV-1 replication and APOBEC3 antiviral activity are not regulated by P bodies Journal of virology 2012 86 11712 11724 10.1128/JVI.00595-12 22915799 PMC3486339

[B49] Goujon C Schaller T Galao RP Amie SM Kim B Olivieri K Neil SJ Malim MH Evidence for IFNalpha-induced, SAMHD1-independent inhibitors of early HIV-1 infection Retrovirology 2013 10 23 10.1186/1742-4690-10-23 23442224 PMC3598776

[B50] Tansey WP Detection of ubiquitylated proteins in mammalian cells CSH protocols 2006 6 10.1101/pdb.prot4616 22485991

