# Supplementary material for: Nuclear import of SAMHD1 is mediated by a classical karyopherin α/β1 dependent pathway and confers sensitivity to VpxMAC induced ubiquitination and proteasomal degradation
Source: Retrovirology. 2014 Apr 8;11:29. doi: 10.1186/1742-4690-11-29 (PMC4098787; doi:10.1186/1742-4690-11-29)
Supplement: Additional file 1 — Contains supplementary Table S1 and supplementary figures S1-S10 with supporting data to the main figures. [file 1742-4690-11-29-S1.pdf]

Table S1

ID	shRNA 1 target sequence 5’-3’	shRNA 2 target sequence 5’-3’
**KPNA1**	GCAGTATGGGCTTTGTCTA	GGAAGAAGGACTGCAGTTA
**KPNA2**	TACTCAAGCTGCCAGGAAA	CCACCAACGAGAATGCTAA
**KPNA3**	GACCCAGGTTGTTCTCAAT	CCACAAGTGATAACCCAGT
**KPNA4**	TCGAAATCCACCAATTGAT	ACCTTACTGATGCTGGCAA
**KPNA5**	GCCCTAAATCCTCAAGAGA	GAAGCAGCTTGGGCTATAA
**KPNA6**	CCCTGAAGAAATGAGACGA	AGCCTAGTCCTCCAATAGA
**KPNB1**	GGCTGAAGCTGCTTATGAA	GTTGCAGCTGGTCTACAAA
**Control**	TCGGCGCAGTCTAATTATA	n.a.

Fig. S1

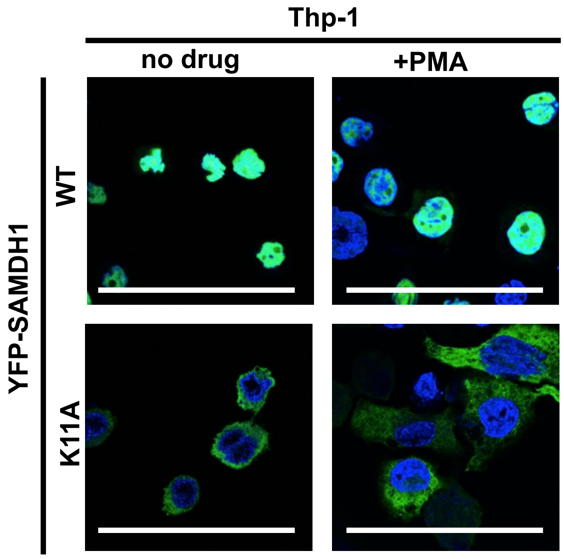

Fig. S2

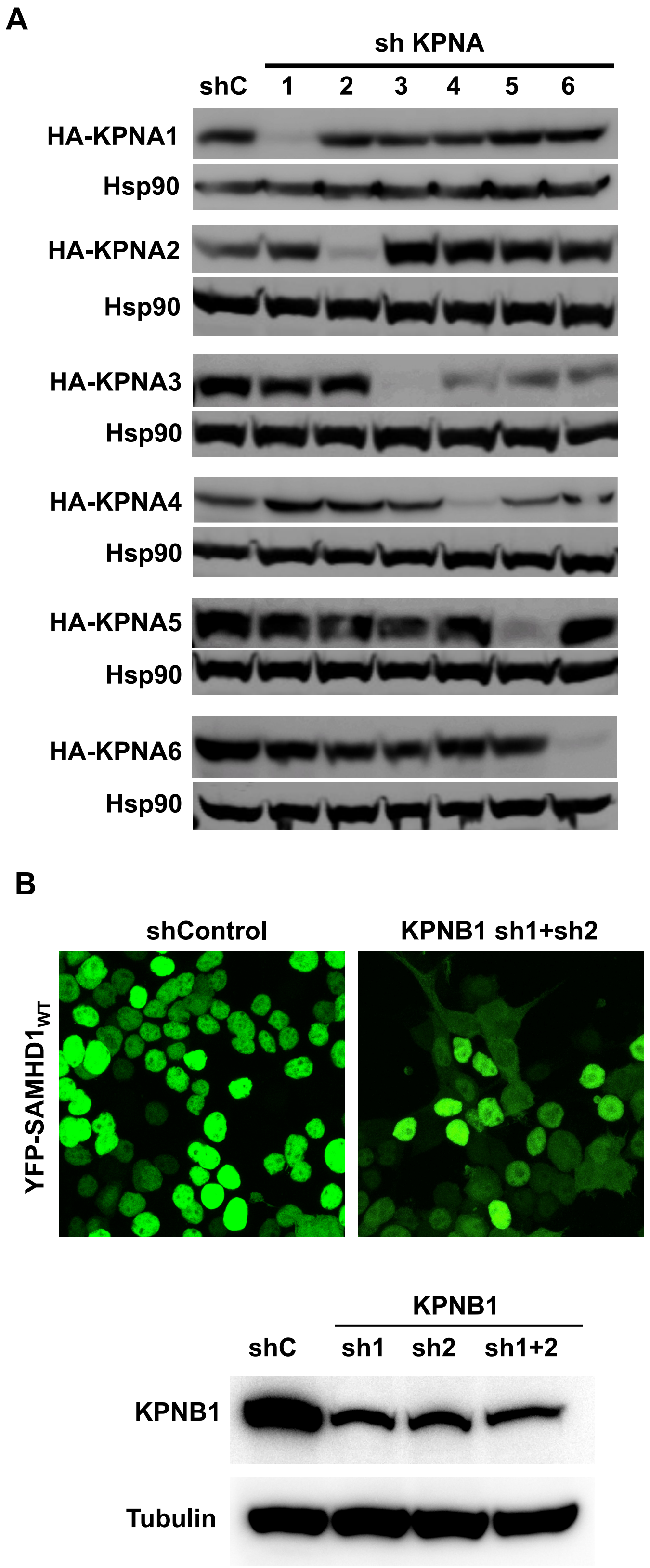
A
sh KPNA
shC
1
2
3
4
5
6
HA-KPNA1
Hsp90
HA-KPNA2
Hsp90
HA-KPNA3
Hsp90
HA-KPNA4
Hsp90
HA-KPNA5
Hsp90
HA-KPNA6
Hsp90
B
shControl
KPNB1 sh1+sh2
YFP-SAMHD1WT
KPNB1
shC
sh1
sh2
sh1+2
KPNB1
Tubulin

Fig. S3

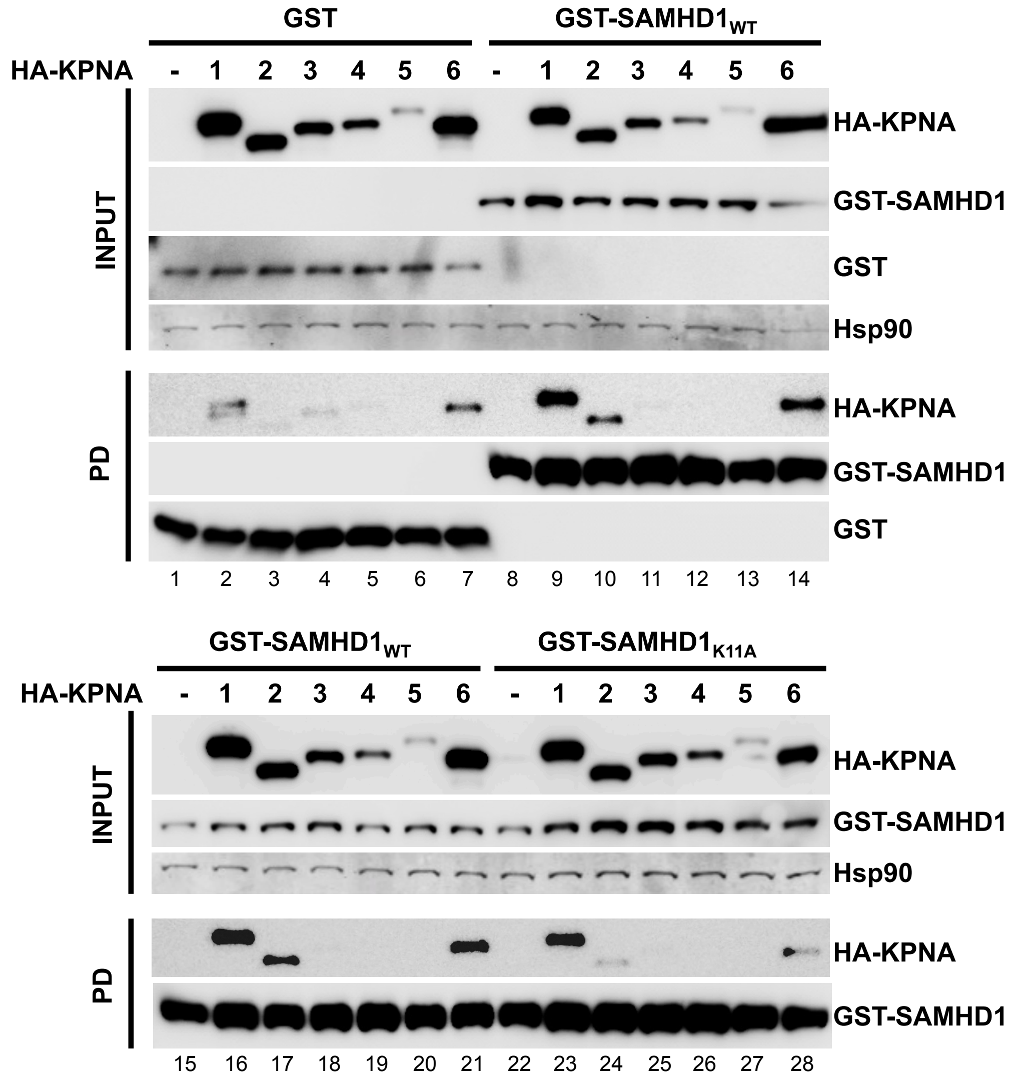
GST
GST-SAMHD1WT
HA-KPNA - 1 2 3 4 5 6 - 1 2 3 4 5 6
INPUT
PD
HA-KPNA
GST-SAMHD1
GST
Hsp90
HA-KPNA
GST-SAMHD1
GST
1 2 3 4 5 6 7 8 9 10 11 12 13 14
GST-SAMHD1WT
GST-SAMHD1K11A
HA-KPNA - 1 2 3 4 5 6 - 1 2 3 4 5 6
INPUT
PD
HA-KPNA
GST-SAMHD1
Hsp90
HA-KPNA
GST-SAMHD1
15 16 17 18 19 20 21 22 23 24 25 26 27 28

Fig. S4

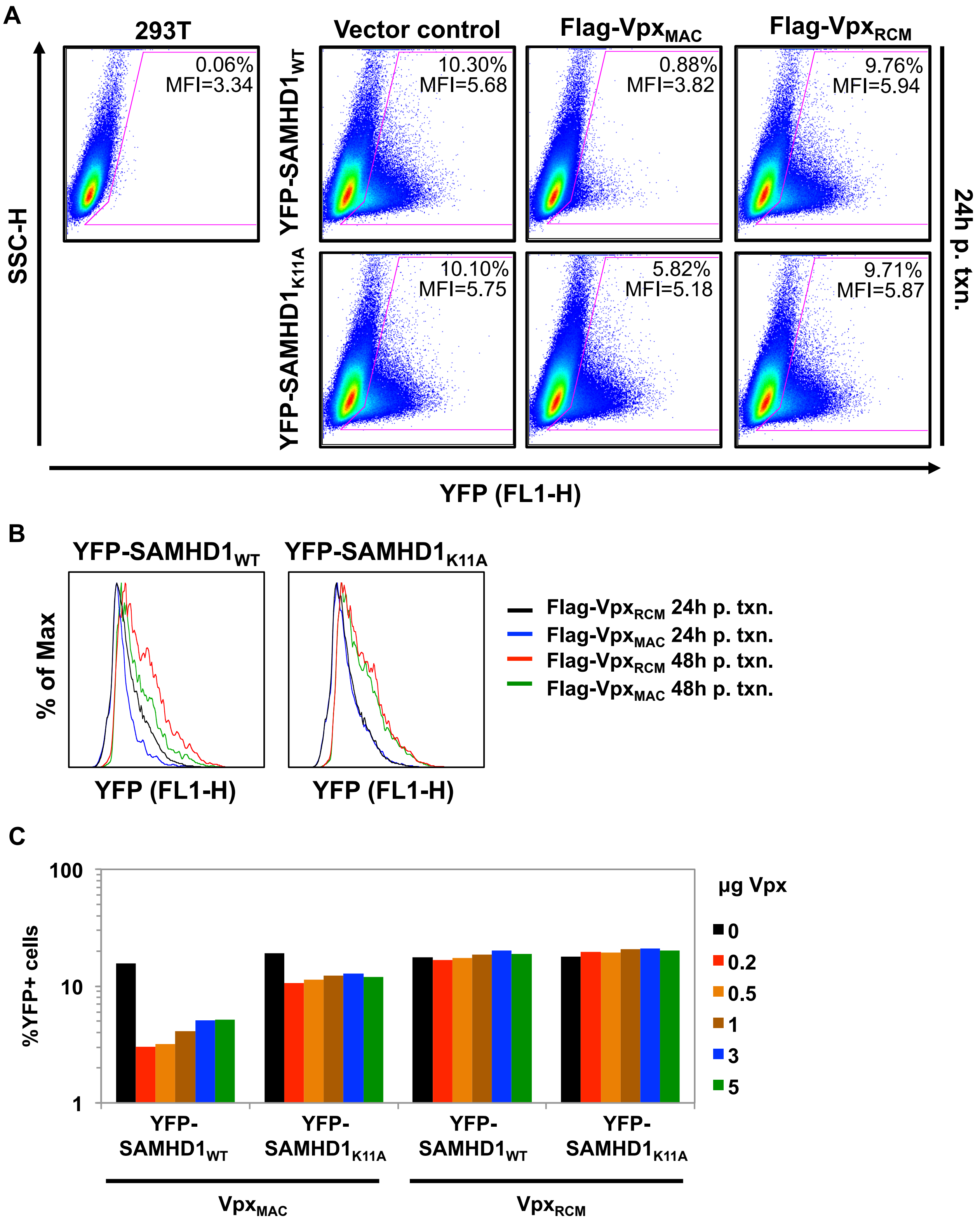

A
293T
Vector control
Flag-Vpx$_{MAC}$
Flag-Vpx$_{RCM}$
0.06%
MFI=3.34
10.30%
MFI=5.68
0.88%
MFI=3.82
9.76%
MFI=5.94
10.10%
MFI=5.75
5.82%
MFI=5.18
9.71%
MFI=5.87
YFP-SAMHD1$_{WT}$
YFP-SAMHD1$_{K11A}$
SSC-H
YFP (FL1-H)
24h p. txn.
B
YFP-SAMHD1$_{WT}$
YFP-SAMHD1$_{K11A}$
% of Max
YFP (FL1-H)
YFP (FL1-H)
Flag-Vpx$_{RCM}$ 24h p. txn.
Flag-Vpx$_{MAC}$ 24h p. txn.
Flag-Vpx$_{RCM}$ 48h p. txn.
Flag-Vpx$_{MAC}$ 48h p. txn.
C
100
10
1
%YFP+ cells
µg Vpx
0
0.2
0.5
1
3
5
YFP-SAMHD1$_{WT}$
YFP-SAMHD1$_{K11A}$
YFP-SAMHD1$_{WT}$
YFP-SAMHD1$_{K11A}$
Vpx$_{MAC}$
Vpx$_{RCM}$

Fig. S5

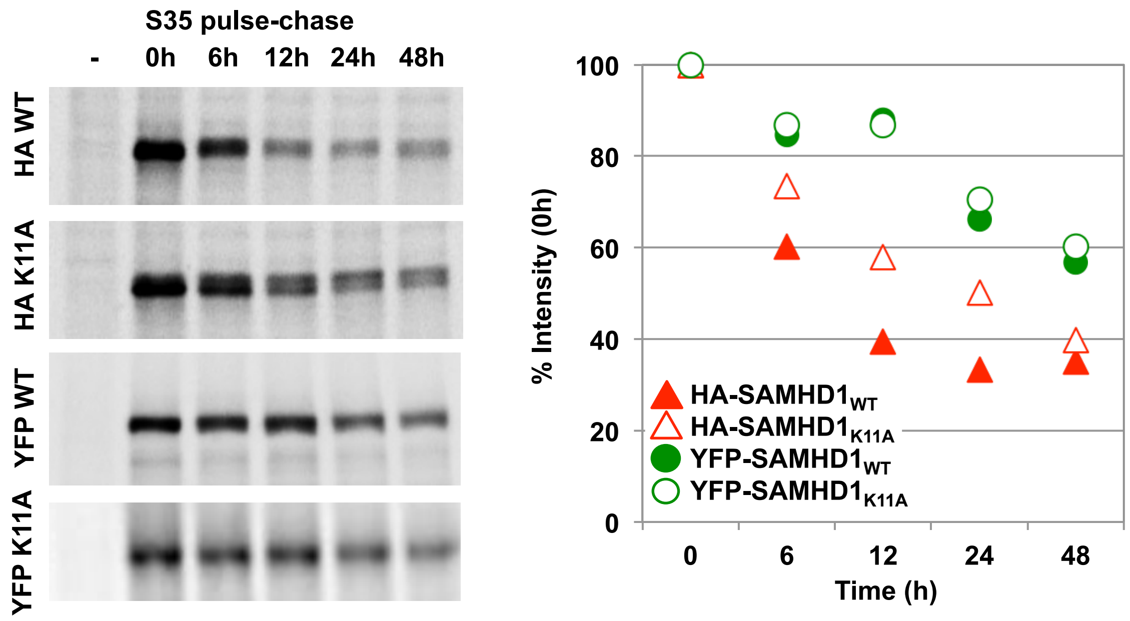

S35 pulse-chase
-
0h
6h
12h
24h
48h
HA WT
HA K11A
YFP WT
YFP K11A
% Intensity (0h)
100
80
60
40
20
0
0
6
12
24
48
Time (h)
HA-SAMHD1WT
HA-SAMHD1K11A
YFP-SAMHD1WT
YFP-SAMHD1K11A

Fig. S6

A

Species	Motif
Homo sapiens	KRPR
Pan troglodytes	....
Pongo abelii	....
Gorilla gorilla	....
Hylobates agilis	....
Macaca mulatta	....
Nomascus leucogenys	....
Callicebus molloch	....
Nasalis larvatus	....
Lagothrix lagotricha	....
Callithrix jacchus	....
Alenopithecus nigroviridis	....
Erythrocebus patas	..L.
Chlorocebus tantalus	..LH
Chlorocebus pygerythrus	..L.
Aotus trivirgatus	....
Cercopithecus neglectus	....
Pteropus vampyrus	.KC.
Myotis lucifugus	....
Echinops telfairi	..A.
Monodelphis domestica	..A.
Erinaceus europaeus	N...
Anolis carolinensis	..AC
Gallus gallus	..A.
Rattus norvegicus	..S.
Mus musculus	....
Oryctolagus cuniculus	...G
Danio rerio	....
Felis catus	....
Canis familiaris	....
Ailuropoda melanoleuca	....
Tursiops truncatus	....
Ochotona princeps	....
Dipodomys ordii	....
Otolemur garnettii	....
Microcebus murinus	....
Equus caballus	....
Bos taurus	....
Sorex araneus	....
Macropus eugenii	..A.

B

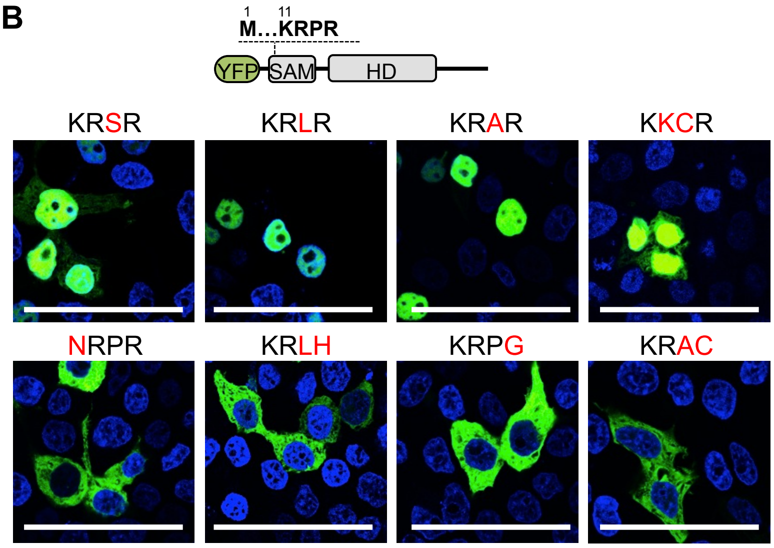

C

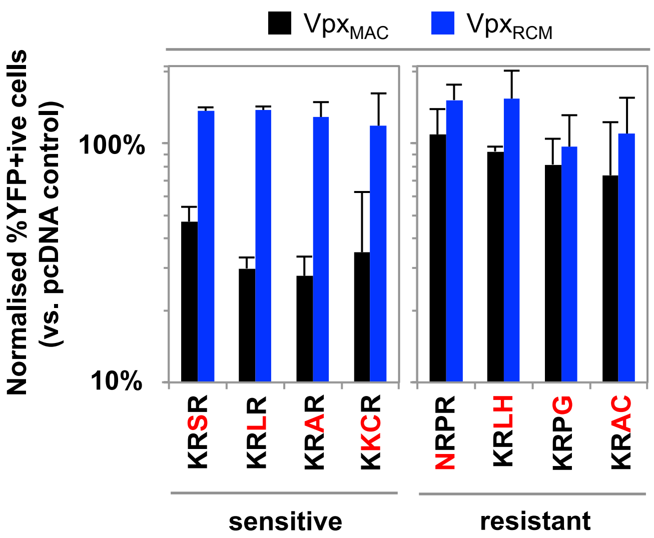

Fig. S7

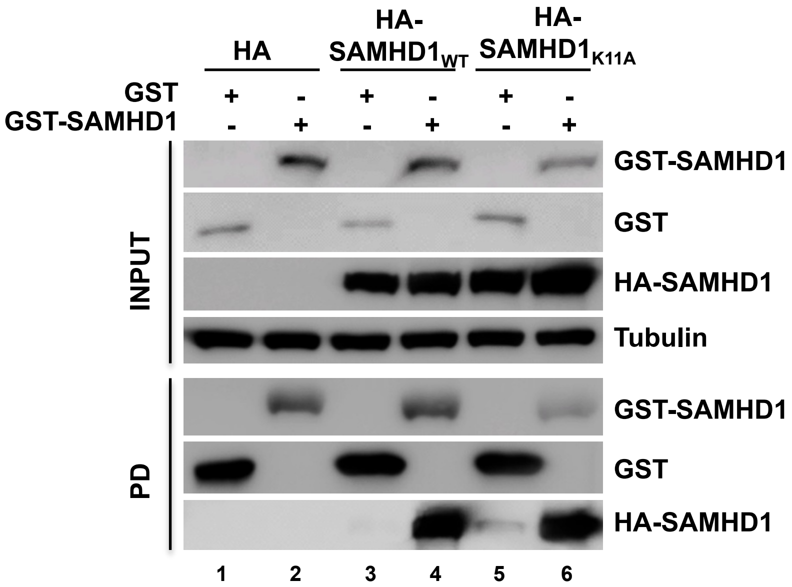
HA
HA-SAMHD1$_{WT}$
HA-SAMHD1$_{K11A}$
GST + - + - + -
GST-SAMHD1 - + - + - +
INPUT
PD
GST-SAMHD1
GST
HA-SAMHD1
Tubulin
GST-SAMHD1
GST
HA-SAMHD1
1 2 3 4 5 6

Fig. S8

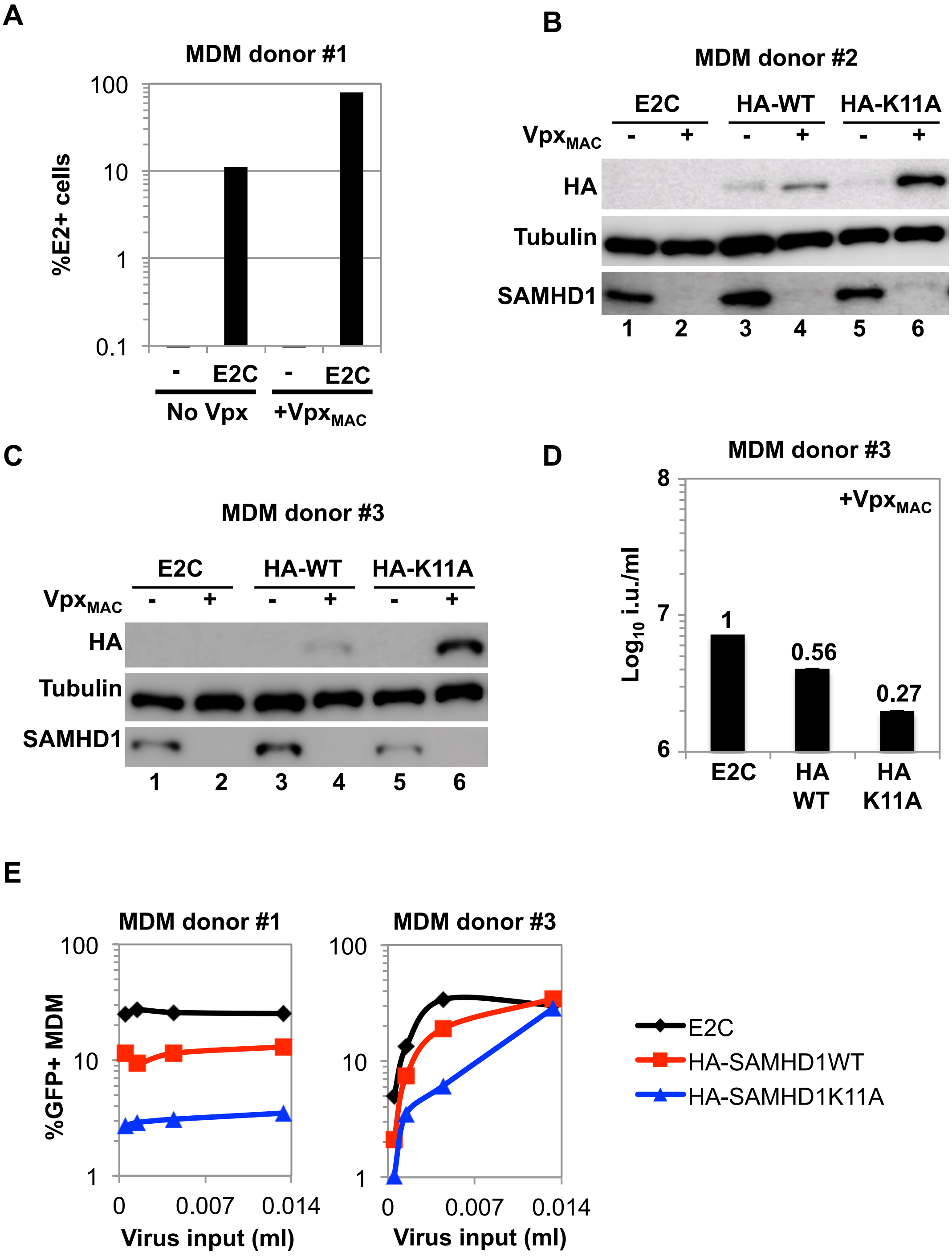

A
MDM donor #1
%E2+ cells
100
10
1
0.1
-
E2C
-
E2C
No Vpx
+Vpx$_{MAC}$
B
MDM donor #2
E2C
HA-WT
HA-K11A
Vpx$_{MAC}$
- + - + - +
HA
Tubulin
SAMHD1
1 2 3 4 5 6
C
MDM donor #3
E2C
HA-WT
HA-K11A
Vpx$_{MAC}$
- + - + - +
HA
Tubulin
SAMHD1
1 2 3 4 5 6
D
MDM donor #3
+Vpx$_{MAC}$
Log_{10} i.u./ml
8
7
6
1
0.56
0.27
E2C
HA WT
HA K11A
E
MDM donor #1
MDM donor #3
%GFP+ MDM
100
10
1
0
0.007
0.014
Virus input (ml)
E2C
HA-SAMHD1WT
HA-SAMHD1K11A

Fig. S9

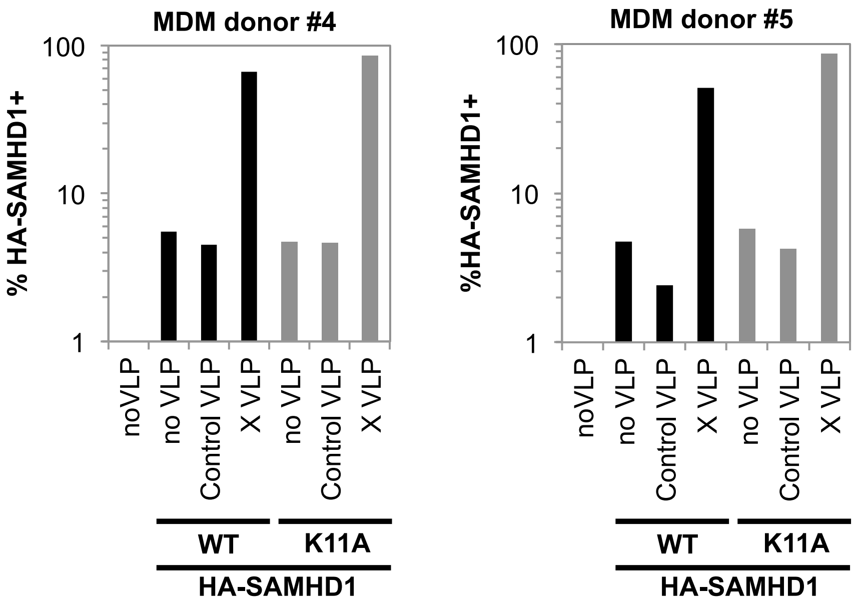
MDM donor #4
% HA-SAMHD1+
100
10
1
noVLP
no VLP
Control VLP
X VLP
no VLP
Control VLP
X VLP
WT
K11A
HA-SAMHD1
MDM donor #5
%HA-SAMHD1+
100
10
1
noVLP
no VLP
Control VLP
X VLP
no VLP
Control VLP
X VLP
WT
K11A
HA-SAMHD1

Fig. S10

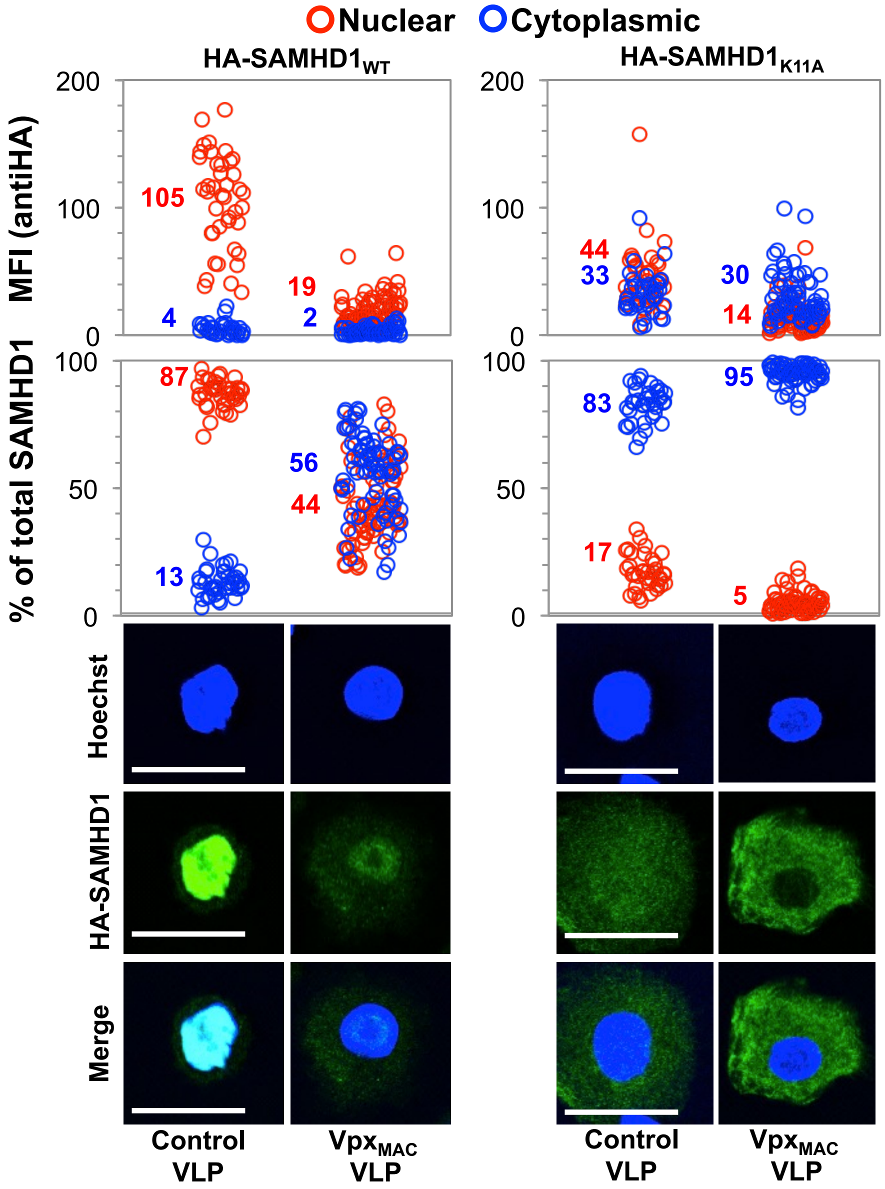

Nuclear
Cytoplasmic
HA-SAMHD1WT
HA-SAMHD1K11A
MFI (antiHA)
200
100
0
105
4
19
2
44
33
30
14
% of total SAMHD1
100
50
0
87
13
56
44
83
17
95
5
Hoechst
HA-SAMHD1
Merge
Control VLP
VpxMAC VLP
Control VLP
VpxMAC VLP
